# Evaluation of pulmonary and systemic toxicity following lung exposure to graphite nanoplates: a member of the graphene-based nanomaterial family

**DOI:** 10.1186/s12989-016-0145-5

**Published:** 2016-06-21

**Authors:** Jenny R. Roberts, Robert R. Mercer, Aleksandr B. Stefaniak, Mohindar S. Seehra, Usha K. Geddam, Ishrat S. Chaudhuri, Angelos Kyrlidis, Vamsi K. Kodali, Tina Sager, Allison Kenyon, Suzan A. Bilgesu, Tracy Eye, James F. Scabilloni, Stephen S. Leonard, Natalie R. Fix, Diane Schwegler-Berry, Breanne Y. Farris, Michael G. Wolfarth, Dale W. Porter, Vincent Castranova, Aaron Erdely

**Affiliations:** 1 National Institute for Occupational Safety and Health, CDC/NIOSH/HELD, 1095 Willowdale Rd., MS4020, Morgantown, WV 26505 USA; 2 West Virginia University, Morgantown, WV 26505 USA; 3 Cabot Corporation, Billerica, MA 01821 USA

**Keywords:** Graphene-based nanomaterials, Pulmonary exposure, Cardiovascular toxicity, Lung toxicity, Particle size

## Abstract

**Background:**

Graphene, a monolayer of carbon, is an engineered nanomaterial (ENM) with physical and chemical properties that may offer application advantages over other carbonaceous ENMs, such as carbon nanotubes (CNT). The goal of this study was to comparatively assess pulmonary and systemic toxicity of graphite nanoplates, a member of the graphene-based nanomaterial family, with respect to nanoplate size.

**Methods:**

Three sizes of graphite nanoplates [20 μm lateral (Gr20), 5 μm lateral (Gr5), and <2 μm lateral (Gr1)] ranging from 8–25 nm in thickness were characterized for difference in surface area, structure,, zeta potential, and agglomeration in dispersion medium, the vehicle for in vivo studies. Mice were exposed by pharyngeal aspiration to these 3 sizes of graphite nanoplates at doses of 4 or 40 μg/mouse, or to carbon black (CB) as a carbonaceous control material. At 4 h, 1 day, 7 days, 1 month, and 2 months post-exposure, bronchoalveolar lavage was performed to collect fluid and cells for analysis of lung injury and inflammation. Particle clearance, histopathology and gene expression in lung tissue were evaluated. In addition, protein levels and gene expression were measured in blood, heart, aorta and liver to assess systemic responses.

**Results:**

All Gr samples were found to be similarly composed of two graphite structures and agglomerated to varying degrees in DM in proportion to the lateral dimension. Surface area for Gr1 was approximately 7-fold greater than Gr5 and Gr20, but was less reactive reactive per m^2^. At the low dose, none of the Gr materials induced toxicity. At the high dose, Gr20 and Gr5 exposure increased indices of lung inflammation and injury in lavage fluid and tissue gene expression to a greater degree and duration than Gr1 and CB. Gr5 and Gr20 showed no or minimal lung epithelial hypertrophy and hyperplasia, and no development of fibrosis by 2 months post-exposure. In addition, the aorta and liver inflammatory and acute phase genes were transiently elevated in Gr5 and Gr20, relative to Gr1.

**Conclusions:**

Pulmonary and systemic toxicity of graphite nanoplates may be dependent on lateral size and/or surface reactivity, with the graphite nanoplates > 5 μm laterally inducing greater toxicity which peaked at the early time points post-exposure relative to the 1–2 μm graphite nanoplate.

**Electronic supplementary material:**

The online version of this article (doi:10.1186/s12989-016-0145-5) contains supplementary material, which is available to authorized users.

## Background

Graphene is chemically defined as a single-atom thick layer of monocrystalline graphite, a two-dimensional sheet of sp^2^-bonded carbon atoms arranged in a hexagonal pattern. Following the first isolation and characterization of graphene [1], research and production of “graphene-related” nanomaterials (one external dimension in the nanoscale) has expanded exponentially [2]. The total forecasted global market for graphene-based materials is tens of billions and hundreds of billions of dollars for large area graphene and bulk or flake graphene, respectively. Applications are led by the field of electronics for large area graphene and use in filtration by both the petroleum and gas industry and the water/wastewater treatment industry for bulk/flake graphene [2]. The global market is predicted to grow considerably in the next 10 years. The current and potential applications of graphene nanomaterials are numerous including, but not limited to, use in electronics, energy storage, lighting, filtration, aerospace engineering, sensors, structural engineering composites, and health [2].

The unique physical and chemical properties of graphene-based nanomaterials are the driving force behind the rapid development and manufacturing of the material [2, 3]. It is highly conductive, both electrically and thermally, reactive to chemicals (“chemically tuned”), has a high surface to weight ratio, and high curability for its density. It is currently the thinnest material ever made, is the most “stretchable” crystal ever made, and is extremely strong. Because of their graphitic nature, graphenes share some of the same industrially advantageous properties as carbon nanotubes (CNTs), including strength, conductivity, and density. In many instances it is considered to be superior to CNT. These factors include increased surface area lending to increased conductivity, decreased aspect ratio for less viscous resins which avoid entanglement issues encountered with CNT, ease of access to precursor material, and a more cost effective production and scalability, all of which may lead to its replacement of CNT in a number of applications [2]. With this growth comes a risk for human exposure to the material along its lifecycle of manufacturing, incorporation into products, use of products, and end of life disposal. The size and density properties of the graphene nanomaterial family, along with known pulmonary toxicity associated with other graphitic nanomaterials, such as CNT, present a concern for respiratory exposure to these materials, particularly in the workplace where the material is handled in powder form prior to incorporation into a final product.

To date, there are a limited number of graphene nanomaterial toxicology studies in vivo, and only a small number of these focus on the respiratory route of exposure [4–11]. To further complicate the interpretation and comparison of in vitro and in vivo studies, terminology related to graphitic nanomaterials has not been consistent. The term “graphene” in industrial and toxicological settings has come to refer to a family of materials that includes any highly exfoliated graphite product [2]. This family includes, but is not limited to, single layer graphene, multi-layer and few-layer graphene, graphite nanosheets/graphite nanoplates/graphite nanoflakes (ultrathin or ultrafine graphite), graphene nanosheets, graphene microsheets, graphene nanoribbons, graphene oxide, graphite oxide, and reduced graphene oxide. An effort has been made to set forth a recommended nomenclature to better define these materials in terms of their physical/chemical properties to remediate the issue [12, 13].

The current study investigated pulmonary and systemic toxicity following pulmonary exposure to graphite nanoplates or nanoplates, as defined by Bianco et al. [12]. Graphite nanoplates can have various industrial applications driven by different lateral dimensions. We hypothesize that different lateral dimensions of graphite nanoplates will confer different toxicity. In the development of structure activity relationship (SAR) models, there is an increasing need for studies that delineate the role of various physico-chemical properties in particle toxicity. There are currently no in vivo comparative toxicity studies on graphene that have evaluated the role of lateral dimension in graphene toxicity. Mice were exposed by a single oropharyngeal aspiration to one of three different sizes of graphite nanoplates suspended in a physiological dispersion medium [14] at a dose of 4 or 40 μg per mouse, dispersion medium control, or carbon black at a dose of 40 μg as a carbonaceous particle control. Parameters of lung injury, inflammation, and oxidative stress, were assessed in lung tissue and bronchoalveolar lavage (BAL) fluid at 4 h, 1 day, 7 days, 1 month, and 2 months post-exposure. Lung histopathology and particle deposition and clearance were also evaluated. Systemic response to lung exposure was assessed as alterations in protein and gene expression in serum, whole blood, heart, aorta, and liver at the same pulmonary recovery time points. Results of the study indicate that the graphite nanoplates with larger lateral dimensions induced a greater level of toxicity in the lung and systemically following exposure when compared to smaller graphite nanoplates, and responses showed resolution over time.

## Results

### Particle characterization

#### Structural analysis

**Fig. 1:**
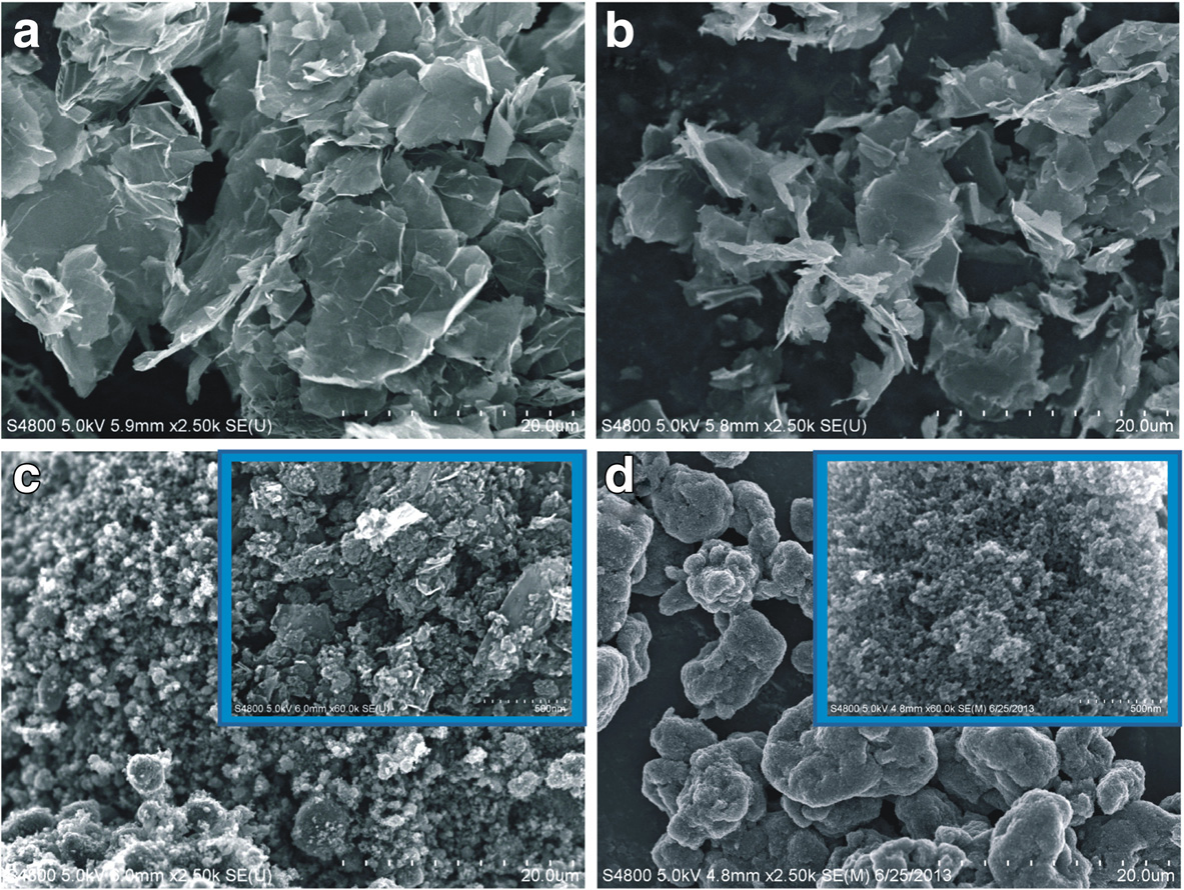
FESEM Images Illustrating Primary Particle Size. FESEM images of dry powder samples of Gr20 (**a**), Gr5 (**b**), Gr1 (**c**), and CB (**d**) at the same magnification illustrating the difference in size of the primary particle. **c** Gr1 Inset: High magnification of the Gr1 sample showing the flake-like structure of the particle similar to that of Gr20 and Gr5, but on a much smaller scale. **d** CB Inset: High magnification of CB shows the primary particles at ~ 20 nm diameter

**Fig. 2:**
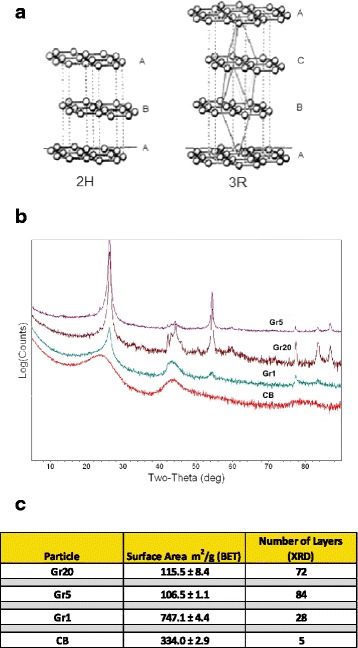
XRD and BET Analysis of Gr and CB Samples. **a** Crystaline structures of Gr samples in this study consisted of two graphitic structures: hexagonal (2H) and rhombohedral (3R), where ABA and ABCA represent stacked graphitic layers. **b** XRD pattern of Gr20 with the Bragg lines identified with 2H, 3R, and 2H/3R phases of graphite. Gr5 and Gr1 had similar patterns, with slightly less pronounced peaks for Gr1. The XRD pattern of CB consist of broad peaks near 2-theta = 24^o^ and 44^o^ typical of disordered carbons. **c** Table summary of surface area of dry powder samples assess using the BET method and the number of layers/sheets in each sample determined by XRD

Field emission scanning electron microscopy (FESEM) was employed to visualize the lateral dimension of the primary particle size of the samples in dry powder form (Fig. 1). The samples were manufactured to have primary particle dimensions of ~ 20 μm lateral (Gr20; Fig. 1a), 5 μm lateral (Gr5; Fig. 1b), and < 2 μm lateral (Gr1; Fig. 1c). Carbon black (CB; Fig. 1d) primary particles were ~20 nm in diameter. X-ray diffraction (XRD) analysis revealed that Gr samples in this study were similar in structure and consisted of two graphitic structures: hexagonal (2H) and rhombohedral (3R), where ABA and ABCA represent stacked graphitic layers (Fig. 2a&b). The graphitic pattern for CB could not be resolved, with no identifiable peaks. Graphite layers in the Gr samples were inversely related to surface area (Fig. 2c). Gr20 and Gr5 were similar in number of layers and surface area, with the lowest surface area, ~ 3-fold lower than CB and 7-fold lower than Gr1. A single layer of unoxidized graphene is 0.34 nm in thickness [15] therefore the average nanoplate thicknesses were calculated to be ~21 nm, ~25 nm and ~8 nm for Gr20, Gr5 and Gr1, respectively, based on the number of graphene layers quantified by XRD. These average thicknesses were greater than what would be predicted by a geometric analysis of the surface area.

#### Agglomeration, stability, and surface impurity/reactivity in DM

**Fig. 3:**
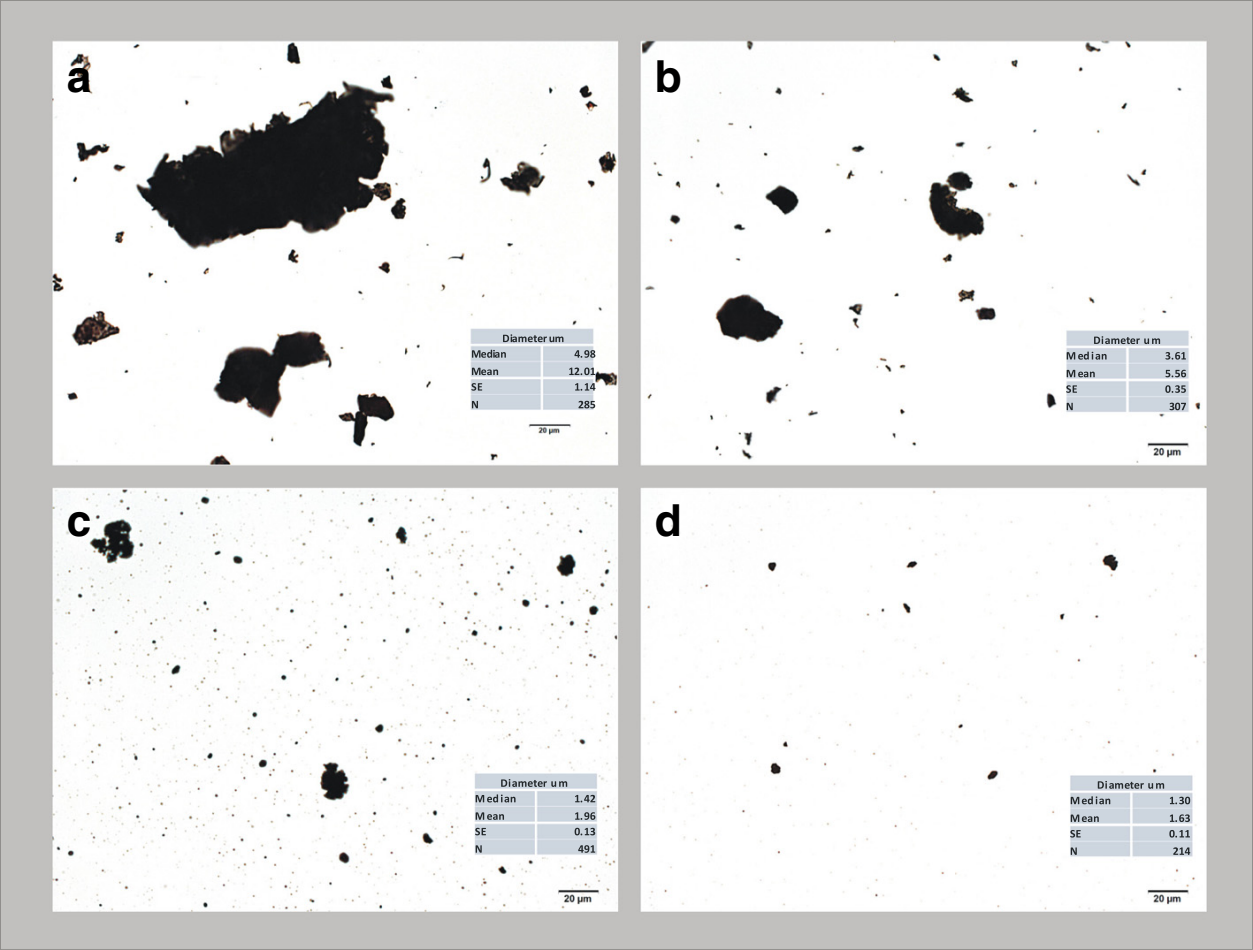
Particle Agglomeration. Light micrographs of the high dose aspiration preparations (0.8 mg/ml DM) of Gr20 (**a**), Gr5 (**b**), Gr1 (**c**), and CB (**d**). Table Insets: Corresponding agglomerate/particle diameter size distributions made from point count measurements on the light microscope (*n*= 214-491 measures). The average cross sectional measures of the agglomerates/particles were 12.01, 5.56, 1.96, and 1.63 μm for Gr20, Gr5, Gr1, and CB, respectively. Agglomerate size ranged up to 300 μm and 60 μm for the Gr20 and Gr5 samples, respectively. Additionally, dynamic light scattering (DLS) was used to measure agglomerate size distribution for Gr1 and CB, resulting in a range of 0.222-2.035 μm for Gr1 and 0.146-2.172 μm for CB. Gr20 and Gr5 did not remain in suspension long enough to accurately size agglomerates using DLS

Agglomeration in dispersion medium (DM; in vivo delivery vehicle) was measured using morphometric point count measurements. Figure 3 illustrates the average agglomerate distributions of the samples prepared for in vivo delivery. The average cross sectional measures of the agglomerates/particles were 12.01, 5.56, 1.96, and 1.63 μm for Gr20, Gr5, Gr1, and CB, respectively. Agglomerate size ranged up to 300 μm and 60 μm for the Gr20 and Gr5 samples, respectively. Additionally, dynamic light scattering (DLS) was used to measure agglomerate size distribution for Gr1 and CB, resulting in a range of 0.222–2.035 μm for Gr1 and 0.146–2.172 μm for CB. Gr20 and Gr5 agglomerates did not remain in suspension long enough to accurately size and exceeded the size limitation of the DLS. Zeta potential of particles in DM did not differ between samples: CB = −17.4 ± 0.507; Gr1 = −19.9 ± 0.161, Gr5 = −21.3 ± 0.242; and Gr20 = −18.1 ± 0.174 (values are means ± SE), indicative of moderate instability of the agglomerates in all samples in DM.

**Fig. 4:**
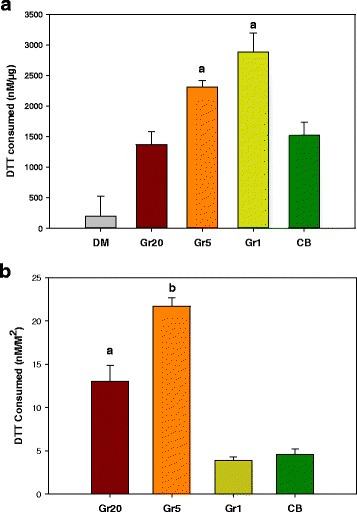
Surface Reactivity by Antioxidant Depletion. Antioxidant (DTT, dithiothreitol) depletion by particles prepared in DM (50 μg/ml) following the same procedures as preparation for aspiration in mice. Data is presented as total DTT consumed on a mass basis (**a**), including the background measurement for the vehicle (DM), and as DTT consumed normalized to surface area (**b**). a- significantly different from the reference material, CB; b- significantly different from all particles; *p* < 0.05

Surface reactivity was assessed via 3 different methods, electron spin resonance (ESR), dichlorofluorescin (DCFH) oxidation assay, and antioxidant (dithiothreitol, DTT) depletion. The generation of radicals on the surface of particles in DM in the presence of hydrogen peroxide as an index of impurities of the surface that can contribute to surface reactivity was measured using electron spin resonance (ESR) and compared to a positive carbonaceous control particle, coal containing α-quartz silica. The generation of radicals by this method was negligible as compared to that of the positive control (data not shown) indicating the particles had very low surface reactivity, and generation of radicals between graphene samples did not differ. Using the dichlorofluorescin (DCFH) assay, although we could see a dose- and time-dependent change in DCFH fluorescence, at all time points measured the fluorescence values from the nanomaterials was lower than what was observed for DM or water control (data not shown). Addition of horseradish peroxidase (HRP) in the reaction mixture amplified the DCFH oxidation and the fluorescence values by 3-fold or higher but did not change the trend that was observed without HRP. The acellular DCFH assay suffered from high background signal resulting from either dye auto-oxidation or as a result of fluorescence quenching by carbonaceous materials. The third cell free assay system, the dithiothreitol (DTT) assay, which is based on antioxidant depletion to determine the oxidative potential of the materials, was performed and data was evaluated in terms of mass of material (Fig. 4a) as well as surface area (Fig. 4b). On a mass basis, Gr1 and Gr5 showed a greater consumption of DTT as compared to Gr20 and CB (Fig. 4a). However, upon normalizing to surface area of the material as measured by BET (Fig. 4b), Gr5 showed the greatest consumption of DTT, followed by Gr20. Both Gr5 and Gr20 showed increased DTT consumption per m^2^ when compared to Gr1 and CB. Gr1 and CB did not differ from each other.

### In vivo studies

#### Deposition/Clearance

**Fig. 5:**
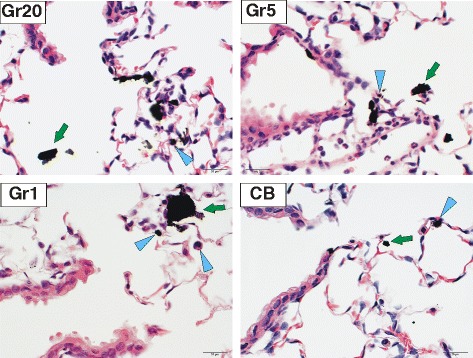
Particle Deposition Following Aspiration. Hematoxylin and eosin-stained lung tissue sections 4 h after mice were exposed to 40 μg of Gr20, Gr5, Gr1, or CB showing differential deposition of particles (Blue arrow heads = particle uptake in macrophages; green arrows = particles in airspace; 60x magnification). All particles were found in both airways and alveoli with majority of all particles located in alveoli (greater than 80 % for all groups), with slight variations in airway deposition depending upon particle size

**Fig. 6:**
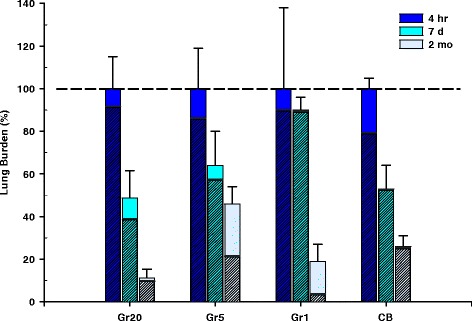
Lung Clearance of Particles. Morphometric analysis of pulmonary clearance of Gr20, Gr5, Gr1, and CB over the 2 month time course assuming 100 % burden at 4 h post-exposure. At 2 months, a greater amount of Gr5 persisted in the lungs when compared to all other particles. The majority of the burden remaining in the lung at 2 months in mice exposed to Gr20 and CB was found to be located primarily in the alveoli (hatched area of bar); whereas Gr5 and Gr1 had greater remaining burden distributed in airways

Particle deposition (Fig. 5) and clearance (Fig. 6) were evaluated morphometrically in animals exposed to the high doses of particles at 4 h following aspiration. At this time point, all particles were found in both lower airways and alveoli with the majority of all particles located in alveoli (greater than 80 %). However, particle deposition in airways varied slightly with particle size, with a slightly greater degree of airway deposition in the Gr5 and CB samples (Fig. 5). Gr20 and Gr1 had a slightly greater deposition in the alveolar region. These differences were not significant. It should be noted that this evaluation may under estimate the total burden, as deposition in large conducting airways was not evaluated. This may be particularly significant for the Gr20 and Gr5 groups as these groups had the larger agglomerate sizes which may have deposited in those larger airways. In general, there tends to be less deposition in these larger airways with the exception of very large agglomerates which would be cleared very quickly. At 2 months, a greater amount of Gr5 persisted in the lungs when compared to all other particles (Fig. 6). The majority of the burden remaining in the lung at 2 months in mice exposed to Gr20 and CB was found to be located primarily in the alveoli; whereas in mice exposed to Gr5 and Gr1, the remaining lung burden was found to be approximately 60 % alveolar in the Gr5 group and 20 % alveolar in the Gr1 group. At all time points post-exposure and in all treatment groups, macrophages recovered by bronchoalveolar lavage (BAL) were observed to contain particles. Images of BAL cells are depicted in Additional file 1: Figure S1 for the 4 h, 7 day, and 2 month time points. In the Gr20 and Gr5 groups, clusters of macrophages were observed in conjunction with the larger agglomerates. At 2 months post-exposure, Gr5 and CB groups appear to have a greater burden per macrophage than the Gr20 and Gr1 groups, which is in agreement with the morphometric analysis of lung clearance. In the Gr20 group, the majority of particles recovered in the BAL were larger agglomerates associated with groups of macrophages, rather than macrophages containing smaller agglomerates as observed in the Gr5 group at that time point. Multinucleated macrophages can be observed in the Gr5 and Gr20 groups at the 2 month time-point; however, these were relatively infrequent and were not quantified as an index of potential frustrated phagocytosis.

#### Histopathology

Histopathological analysis for alveolar interstitial inflammation (AII), bronchiolar inflammation (BI), terminal bronchiolar inflammation (TBI), and bronchiolar hypertrophy or hyperplasia (BHH) was performed at 7 days, 1 month, and 2 months post-exposure for all treatment groups (*n* = 6 per group per time point) (Table 1). The presence of type II epithelial cell hypertrophy, bronchus-associated lymphoid tissue (BALT), lipidosis, and fibrosis was also evaluated. Alterations were scored from 0–5 for severity. No pathological changes were found in animals exposed to DM, low doses of Gr samples, or the high dose of Gr1. On day 7, TBI was noted as minimal in the Gr20 (average score = 1.2), Gr5 (average score = 1), and CB (average score = 0.5) groups. BHH was also scored as minimal in the Gr20 group (average score = 1) at day 7. One animal in the Gr20 group showed BI on day 7. No changes were noted in the CB group at 1 or 2 months. BHH persisted as minimal in the Gr20 group throughout the time course (average score of 0.7 and 0.8 at 1 and 2 months), and was also observed to be minimal in the Gr5 group at 1 and 2 months (average score of 0.5). TBI also persisted as minimal in the Gr20 group with decreased severity over time (average score =0.7 at 2 months). TBI decreased over time in the Gr5 group and was not observed at 2 months. Minimal AII (score = 1) was only observed in 2 animals in the Gr5 group at 2 months. One fibrotic focal area was noted in 1 animal from the Gr20 group at 2 months and was scored as minimal (*n* = 1). No fibrosis, lipidosis, granulomatous formations, type II pneumocyte hypertrophy or development of BALT was noted. Although animals exposed to the larger Gr samples were noted to have a higher degree of inflammation, severity either remained level or decreased over time suggesting no overt disease associated with exposure.

**Table 1:** Histopathology analysis of lung tissue following exposure to 40 μg of particles

Treatment Group	Time Post-Exposure	AII	BI	TBI	BHH
Gr20	7 day	-	**0.17** (1/6)	**1.7** (6/6)	**1.0** (5/6)
Gr5	7 day	-	-	**1.0** (5/6)	-
Gr1	7 day	-	-	-	-
CB	7 day	-	-	**0.5** (3/6)	**0.17** (1/6)
Gr20	1 month	**0.17** (1/6)	-	**0.7** (4/6)	**0.7** (4/6)
Gr5	1 month	-	-	**0.7** (2/6)	**0.5** (3/6)
Gr1	1 month	-	-	-	-
CB	1 month	-	-	-	-
Gr20	2 months	**-**	-	**0.7** (4/6)	**0.83** (5/6)
Gr5	2 months	**0.3** (2/6)	-	-	**0.5** (3/6)
Gr1	2 months	-	-	-	-
CB	2 months	-	-	-	-

Lung tissue was analyzed for alveolar interstitial inflammation (AII), bronchiolar inflammation (BI), terminal bronchiolar inflammation (TBI), and bronchiolar hypertrophy or hyperplasia (BHH), type II cell hypertrophy, BALT, lipidosis, fibrosis (*n* = 6 per group per time point). Severity was scored as 0-5: 0 = normal, 1 = minimal/slight, 2 = mild, 3 = moderate, 4 = marked, and 5 = severe. Data are presented as means in bold with incidence (number of animals with a positive score per total animals) in parentheses. No changes in type II cell hypertrophy, BALT, lipidosis, or fibrosis were noted with the exception of one 1 animal from the Gr20 group at 2 months that had one fibrotic focal area scored as 1

#### Bronchoalveolar Lavage (BAL) analysis

**Fig. 7:**
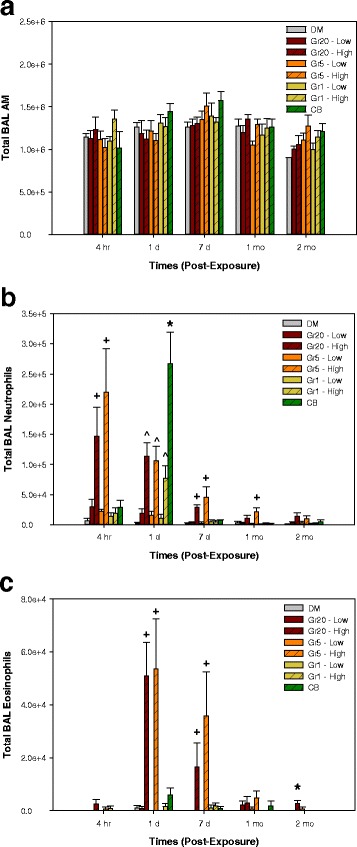
Cellular Differentials in Bronchoalveolar Lavage. Total BAL cells recovered from mice 4 h, 1 day, 7 days, 1 month, and 2 months post-exposure to 4 or 40 μg of Gr20, Gr5, or Gr1, 40 μg of CB, or DM alone differentiated into AM (**a**), neutrophils (**b**), and eosinophils (**c**) (lymphocytes not shown). *significantly different from all groups; ^+^ significantly different from DM, Gr20-Low, Gr5-Low, Gr1-Low, Gr1-High, and CB; ^^^significantly different from DM, Gr20-Low, Gr5-Low, and Gr1-Low; *p* < 0.05

Cell counts and differentials were performed on cells recovered by BAL at 4 h, 1 day, 7 days, 1 month and 2 months post-exposure (Fig. 7). Neutrophil influx was greatest in the CB reference particle group relative to all other groups at 1 day post-exposure, but was not increased in the CB group relative to DM at any other time point (Fig. 7b). Gr1 also had an increase in neutrophil influx only at 1 day post-exposure, a similar pattern to CB albeit to a lesser degree. Exposure to the larger sizes of graphite nanoplates (Gr20 and Gr5) at the high dose significantly increased neutrophils in the lungs up to 1 month post-exposure, peaking at the 4 h time point and decreasing over time relative to control and to the low dose groups (Fig. 7b Increases in eosinophils followed a similar pattern as neutrophil influx peaking on 1 day post-exposure for the Gr5 and Gr20 groups (Fig. 7c); however, no increase in eosinophils was found in the CB or Gr1 groups. A non-significant increase in lymphocytes was observed only at 7 days post-exposure in the Gr5 and Gr20 group (data not shown), and there were no significant differences in macrophage influx (Fig. 7a) into the lungs of treated mice.

**Fig. 8:**
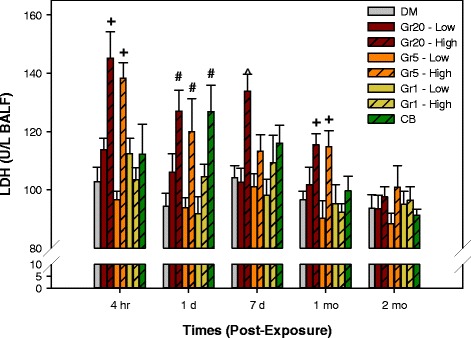
Lung Injury. Lactate dehydrogenase (LDH) activity, an indicator of cytotoxicity, in BALF recovered from mice 4 h, 1 day, 7 days, 1 month, and 2 months post-exposure to 4 or 40 μg of Gr20, Gr5, or Gr1, 40 μg of CB, or DM alone. ^+^significantly different from DM, Gr20-Low, Gr5-Low, Gr1- Low, Gr1-High, and CB; ^#^significantly different from DM, Gr5-Low, and Gr1-Low; ^^^significantly different from DM, Gr20-Low, Gr5-Low, and Gr1-Low; *p* < 0.05

BAL fluid (BALF) was analyzed for the presence of lactate dehydrogenase (LDH) activity as an index of cytotoxicity and lung injury (Fig. 8). Exposure to the larger sizes of graphite nanoplates (Gr20 and Gr5) at the high dose increased LDH at 4 h and 1 day post-exposure. Although injury was still significantly increased at 1 month post-exposure, LDH activity levels began decreasing over time after 1 day, and had returned to control levels by 2 months, indicating resolution of lung injury over time.

A panel of 60 proteins (Additional file 2: Table S1) in BALF was measured reflecting the activity of cellular pathways including chemotaxis, inflammation, oxidative stress, tissue remodeling, and immune response. No significant differences were observed in the low dose groups at 1 day post-exposure, and proteins in the low dose groups were, consequently, not analyzed at the later time points post-exposure. At 1 day post-exposure, ~40 of the 60 proteins measured in the BALF, related primarily to inflammation, acute phase response, chemotaxis, and tissue remodeling pathways, as well as some immune-regulatory responses, were elevated differentially as follows in animals exposed to the high doses of particles when compared to DM: Gr5 > CB ≥ Gr20 > Gr1 (Table 2). The exception to the rule occurred with interleukin (IL)-5 and tissue inhibitor of metalloproteinase (TIMP)-1, as well as a trend for matrix metalloproteinase (MMP)-9, where Gr20 and Gr5 produced a similar response to each other that was greater than that of the CB group. Levels of all significantly elevated proteins on day 1 decreased over time for all groups to varying degrees. At 7 days and 1 month post-exposure, several proteins remained significantly elevated for Gr5 and Gr20, while no significant changes were evident at 1 month post-exposure for the CB- or Gr1-treated groups. Proteins elevated at 1 month in the Gr20 and Gr5 groups were primarily indicative of inflammation/chemotaxis (macrophage colony stimulating factor, macrophage inflammatory proteins, macrophage –derived chemokine, myeloperoxidase), and tissue remodeling (MMP-9, TIMP-1, and fibrinogen). The data indicate that the larger graphite nanoplates may be producing a greater degree of persistent inflammation and injury in the lung. A similar battery of protein analysis in BALF was conducted for the DM, Gr5 and Gr20 high dose groups only at 2 months post-exposure (Table 3). Overall, protein concentrations in BALF continued to decrease in Gr5 and Gr20 groups over the time course. Although there were a greater number of proteins that remained significantly elevated in the Gr5 group versus the Gr20 group when compared to DM (primarily related to inflammation/chemotaxis), the fold-increase was lower than that of the early time points indicating resolution of most responses. The differences that existed may relate to lung burden at 2 months following exposure, where animals exposed to the high dose of Gr5 had the slowest particle clearance rate with ~60 % of particles persisting in the alveolar region at that time.

**Table 2:** BALF protein analysis at 1 day, 7 days and 1 month

	1 Day				7 Day				1 Month			
Protein	Gr20 - High	Gr5 - High	Gr1 - High	CB - High	Gr20 - High	Gr5 - High	Gr1 - High	CB - High	Gr20 - High	Gr5 - High	Gr1 - High	CB - High
Apo A-1	**3.44** ^**b,c**^	**3.02** ^**b,c**^	1.54	**2.57** ^**b**^	0.98	0.76	0.73	0.48	1.26	1.34	1.24	0.94
CRP Mouse	**1.94** ^**c**^	**1.53** ^**c**^	1.18	**1.29**	1.07	1.07	0.80	1.00	1.29	1.21	1.29	1.07
CD40	BLD	BLD	BLD	**1.36** ^**e**^	BLD	1.63	BLD	1.69	BLD	BLD	BLD	BLD
CD40L	**26.74** ^**c**^	**35.13** ^**c**^	**9.3** ^**b**^	**33.74** ^**c**^	2.41	2.76	0.97	3.04	1.00	1.13	0.62	0.78
Eotaxin	**3.2** ^**c**^	**6.46** ^**c**^	**1.36** ^**b**^	**2.04** ^**c**^	1.46	1.56	1.22	1.22	1.07	1.08	1.10	0.94
EGF Mouse	**2.63** ^**b,c**^	**3.12** ^**c**^	**2.34** ^**b**^	**3.12** ^**c**^	**2.46** ^**a**^	**2.28** ^**a**^	**2.06** ^**a**^	**2.02** ^**a**^	0.84	0.94	0.68	0.68
Factor VII	**1.83** ^**b**^	**2.53** ^**c**^	**1.66** ^**b**^	**2.23** ^**c**^	1.73	1.41	1.47	1.39	1.08	1.11	1.07	0.98
Fibrinogen	**2.77** ^**c**^	**2.35** ^**c**^	1.26	**2.07** ^**c**^	**1.29**	0.94	0.79	1.12	**1.35** ^**a**^	1.28	1.23	1.11
GCP-2 Mouse	**1.83** ^**b**^	**2.70** ^**c**^	**2.94** ^**c**^	**3.17** ^**c**^	**2.12** ^**c**^	**1.71** ^**c**^	**2.27** ^**c**^	1.68	1.04	0.89	0.70	0.97
GM-CSF	**2.39** ^**b**^	**5.75** ^**c**^	**4.49** ^**c**^	**24.48** ^**e**^	2.02	1.36	1.71	1.18	1.15	1.06	0.87	0.92
KC/GRO	**4.17** ^**b**^	**12.5** ^**c**^	**7.08** ^**c**^	**23.33** ^**e**^	BLD	BLD	BLD	BLD	BLD	BLD	BLD	BLD
IgA	0.93	1.64	1.00	1.30	0.86	0.84	0.80	0.65	1.18	1.00	0.99	1.03
IP-10	**10.76** ^**b**^	**14.28** ^**b**^	1.98	**7.59** ^**b**^	1.48	1.58	1.48	2.47	0.84	1.02	0.93	1.02
IL-1α	**1.86** ^**b**^	**2.66** ^**c**^	**1.78** ^**b**^	**3.32** ^**c**^	2.45	3.46	2.62	2.57	1.26	1.55	0.42	1.31
IL-1β	1.26	**2.00**	1.01	**2.08** ^**c**^	0.99	1.03	0.82	0.87	0.72	1.22	1.32	1.12
IL-5	**6.22** ^**d**^	**8.49** ^**d**^	1.26	**2.26** ^**c**^	BLD	BLD	BLD	BLD	BLD	BLD	BLD	BLD
IL-6	**16.34** ^**c**^	**39.02** ^**e**^	**4.65** ^**b**^	**13.99** ^**c**^	BLD	BLD	BLD	BLD	BLD	BLD	BLD	BLD
IL-18	**1.60** ^**b**^	**2.26** ^**c**^	**1.50** ^**b**^	**2.74** ^**c**^	0.99	1.35	0.96	1.10	0.81	1.17	0.92	1.06
LIF	**3.91** ^**c**^	**6.57** ^**d**^	**1.99** ^**b**^	**3.01** ^**c**^	**2**.**2** **5** ^a^	1.81	1.89	1.62	1.00	0.98	0.81	0.87
Lymphotactin	**1.61**	**2.39** ^**c**^	1.14	**1.91**	1.18	1.10	1.17	1.42	0.62	0.81	1.00	1.01
M-CSF-1	**1.62** ^**b**^	**2.31** ^**c**^	**1.62** ^**b**^	**3.91** ^**e**^	1.32	1.22	1.03	1.19	**1.18** ^**c**^	**1.27** ^**c**^	0.97	0.92
MDC	**6.04** ^**c**^	**8.58** ^**e**^	1.78	**4.09** ^**c**^	**2.34** ^**a**^	**1.84** ^**a**^	**1.51** ^**a**^	**1.78** ^**a**^	**1.63** ^**c**^	**1.76** ^**c**^	1.10	**1.36**
MIP-1α	**2.84** ^**c**^	**4.12** ^**d**^	**1.70** ^**b**^	**3.56** ^**d**^	**2.50**	2.32	**2.40**	1.71	**1.82**	**3.33** ^**b**^	1.81	1.50
MIP-1β	**14.51** ^**c**^	**14.99** ^**c**^	**3.09** ^**b**^	**12.94** ^**c**^	1.40	1.79	1.46	2.13	0.82	1.25	0.75	0.74
MIP-1γ	**6.33** ^**c**^	**9.03** ^**c**^	**3.94** ^**b**^	**10.35** ^**c**^	**2.95** ^**a**^	**3.78** ^**a**^	1.38	2.11	**2.75** ^**c**^	**3.69** ^**c**^	1.30	1.69
MIP-2	**4.54** ^**c**^	**5.54** ^**c**^	**2.94** ^**b**^	**5.70** ^**c**^	**2.26** ^**a**^	**1.93**	**1.89**	**1.76**	0.76	0.96	0.60	0.85
MIP-3	**2.34** ^**c**^	**3.56** ^**e**^	**1.78** ^**b**^	**2.50** ^**c**^	**2.13** ^**a**^	**2.08** ^**a**^	**2.15** ^**a**^	**1.95** ^**a**^	1.17	1.09	0.88	1.01
MMP-9	**71.42** ^**c**^	**52.44** ^**c**^	**11.19** ^**b**^	**46.72** ^**c**^	**4.08**	**4.51**	2.46	**4.90**	**2.07** ^**c**^	**1.87** ^**c**^	0.63	0.95
MCP-1	**17.86** ^**c**^	**25.76** ^**c**^	0.98	**15.54** ^**c**^	BLD	BLD	BLD	7.06	BLD	BLD	BLD	BLD
MCP-3	**38.32** ^**c**^	**42.07** ^**c**^	**3.01** ^**b**^	**21.82** ^**c**^	1.18	**3.57** ^**a**^	**1.65** ^**a**^	**5.56** ^**a**^	1.13	**2.04** ^**e**^	0.97	1.05
MCP-5	**9.27** ^**c**^	**10.11** ^**c**^	BLD	**5.38** ^**c**^	BLD	BLD	0.73	1.03	BLD	BLD	BLD	BLD
MPO	**51.27** ^**c**^	**66.02** ^**d**^	**18.32** ^**c**^	**62.77** ^**d**^	**21.78** ^**a**^	**20.26** ^**c**^	**7.23** ^**a**^	**25.24** ^**c**^	**4.68** ^**c**^	**3.58** ^**c**^	0.59	1.01
PAI-1	**1.84** ^**c**^	**2.63** ^**e**^	**1.50** ^**b**^	**1.64** ^**c**^	1.17	1.11	0.98	1.21	1.12	1.37	1.15	1.14
SAP	**2.63** ^**b,c**^	**2.03** ^**b**^	1.09	1.50	1.38	1.00	0.68	0.71	1.00	0.97	1.13	0.63
SCF	**2.36** ^**b**^	**3.37** ^**c**^	1.39	**2.66** ^**c**^	0.88	1.43	1.51	2.09	1.26	0.88	0.82	0.83
Thrombopoetin	**2.53** ^**b**^	**4.17** ^**c**^	**2.20** ^**b**^	**4.42** ^**c**^	1.47	1.03	BLD	BLD	BLD	1.58	BLD	BLD
TIMP-1	**17.32** ^**d**^	**18.88** ^**d**^	**2.95** ^**b**^	**7.49** ^**c**^	1.59	**1.82** ^**a**^	1.12	1.18	**1.37**	**1.53** ^**c**^	**1.30**	1.97
TNF-α	BLD	**3.90** ^**e**^	BLD	BLD	BLD	BLD	BLD	BLD	BLD	BLD	BLD	BLD
VCAM-1	**1.88** ^**c**^	**2.08** ^**c**^	1.30	**1.86** ^**c**^	1.13	1.21	0.84	1.07	1.16	1.20	1.08	0.98
VEGF-A	**2.16** ^**c**^	**2.45** ^**c**^	**1.44** ^**b**^	**2.49** ^**c**^	1.14	1.08	1.04	1.04	0.96	0.98	0.93	0.85
vWF	**1.76** ^**b**^	**2.10** ^**b**^	**1.73** ^**b**^	**2.33** ^**c**^	1.63	1.63	1.52	1.29	1.22	1.11	1.00	1.05

Data in the table represent fold-increase for proteins that were significantly different for one or more groups at one or more time points post-exposure based on RodentMap® v2.0. The low dose groups were tested only at 1 day post-exposure. BLD – below the limit of detection. Groups with like symbols denote statistically similar group; ^a^statistically different from DM only; ^b^statistically different from DM and all low dose groups; ^c^statistically different from DM and at least one high dose group; ^d^statistically different from statistically different from DM, low dose groups, and at least one high dose group; ^e^statistically different from all groups; *p* < 0.05. Bold text without symbols denotes significance at *p* < 0.07

**Table 3:** BALF protein analysis at 2 months post-exposure

Protein	Gr20 - High	Gr5 - High	Protein	Gr20 - High	Gr5 - High
**Apo A-1**	1.53	0.99	**MIP - 1α**	1.33	1.44
**Crp Mouse**	1.00	1.19**	**MIP - 1β**	1.13	1.61**
**Eotaxin**	1.37*	1.52*	**MIP - 1γ**	2.54*	4.08*
**EGF Mouse**	**3.93**	**1.52**	**MIP - 2**	1.65	2.26**
**Fibrinogen**	1.07	1.32	**MIP - 3**	1.36	1.57
**GCP-2 Mouse**	1.37	1.37	**MMP-9**	2.28	**3.47**
**GM-CSF**	BLD	BLD	**MCP-1**	BLD	2.47
**KC/GRO**	BLD	BLD	**MCP-3**	0.58	5.64**
**IgA**	1.05	2.01**	**MCP-5**	BLD	BLD
**IP-10**	1.42	2.11	**PAI-1**	1.02	1.36**
**IL-1α**	0.79	3.16	**SAP**	BLD	BLD
**IL-1** $\boldsymbol{\upbeta}$	BLD	BLD	**SCF**	0.78	0.94
**IL-5**	BLD	BLD	**Thrombopoetin**	1.90	2.52
**IL-6**	**1.12**	2.48**	**TIMP-1**	1.20*	1.37**
**IL-18**	2.31	3.50*	**TNF-α**	BLD	BLD
**LIF**	1.48	1.73*	**VCAM-1**	1.40*	1.46*
**M-CSF-1**	1.13	1.48**	**VEGF-A**	0.74	0.79
**MDC**	2.01*	2.38*	**vWF**	1.41	1.46

Data in the table represent fold-increase for proteins that were significantly different for one or more groups at one or more time points post-exposure based on RodentMap® v3.0 (Does not include CD40, CD40L, Lymphotactin, MPO, and osteopontin). BLD – below the limit of detection; ** -statistically different from Gr20 and DM; *- statistically different from DM; *p* < 0.05. Bold text without symbols denotes significance at *p* < 0.07

#### Relative mRNA expression from lung

**Fig. 9:**
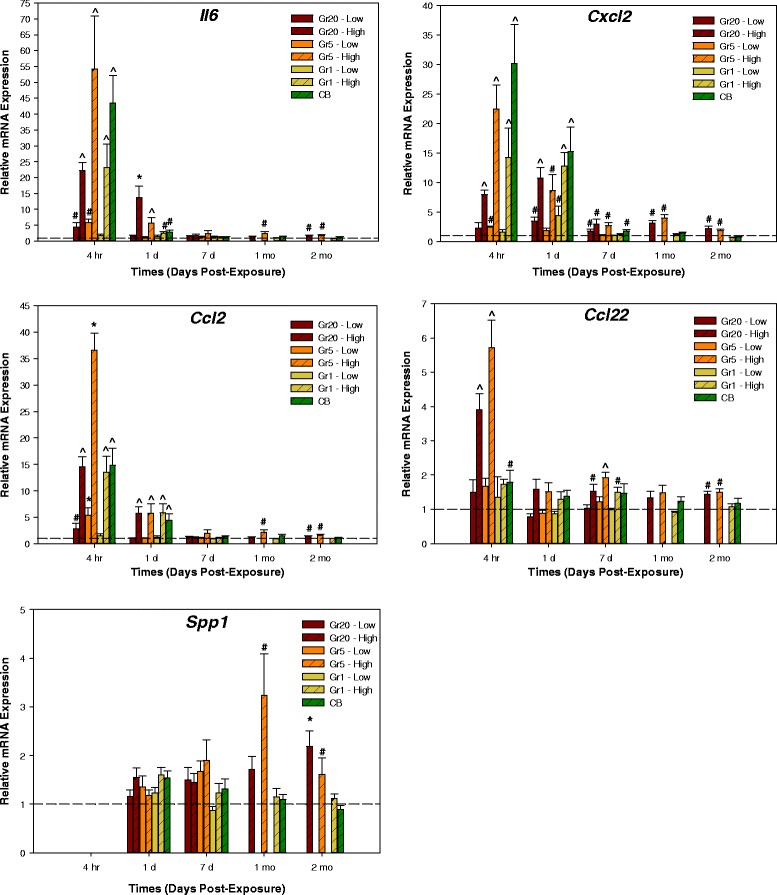
Gene Expression in Lung Tissue. Realtive mRNA expression of IL-6 (*Il6*), MIP-2 (*Cxcl2*), MCP-1 (*Ccl2*), MDC (*Ccl22*), and osteopontin (*Spp1*) relative to DM control at all time points post-exposure, with the exception of *Spp1* which was not measured at 4 h post-exposure. Low dose groups were not evaluated at 1 and 2 months post-exposure. ^*^significantly different from all groups; ^#^ significantly different from DM only; ^^^ significantly different from DM, Gr20-Low, Gr5-Low, and Gr1-Low; *p* < 0.05

A screen of 93 target genes (Additional file 3: Table S2) was analyzed for the lung. Categorically, these genes represent mediators of tissue remodeling, inflammation/acute phase response, immune response, chemotaxis, endothelial cell activation, oxidative stress, coagulation, and cellular lifecycle/apoptosis pathways. At 4 h post-exposure, significant alterations in relative expression of various mediators were observed (Additional file 4: Table S3). These mediators related largely to the inflammation/acute phase and chemotaxis/endothelial cell activation pathways, and several immune response and tissue remodeling genes were also increased, complementing the protein data obtained in the BALF. In general, the effects were greater in the Gr20- and Gr5-exposed mice compared to Gr1. At 1 day post-exposure, similar potency was observed with responses being greater in Gr20- and Gr5-exposed mice compared to Gr1 (Additional file 4: Table S3). In a temporal fashion, the level of response was less at 1 day as compared to 4 h post-exposure. A subset of genes including *Il6, Cxcl2, Ccl2,* and *Ccl22* were further examined and showed a general resolution with time when evaluating all post-exposure time points (Fig. 9). *Il6* represents a primary inflammatory cytokine which stimulates the immune and acute phase response. *Cxcl2* is an indicator of the neutrophil response while *Ccl2* indicates macrophage activation. *Ccl22* also indicates macrophage, and potentially dendritic cell activation. Ccl22 was related to declining lung function following a particulate exposure [16, 17] and persists longer than other traditional mediators [18], hence its inclusion in thepnale of toxicity markers. The effects were more sustained in the Gr20 and Gr5 groups when compared to Gr1 for those four genes. CB was similar to Gr1 in that acute inflammation was observed at early time points with resolution observed by day 7. The low doses of Gr20 and Gr5 showed initial inflammation at 4 h with increased expression of *Il6, Cxcl2,* and *Ccl2* that resolved by 1 day post-exposure. The expression of osteopontin, *Spp1*, which can be suggestive of granuloma production as well as other immune responses in lung tissue, increased with time reaching significance at the later time points for Gr20 and Gr5.

#### Screen for systemic responses

**Fig. 10:**
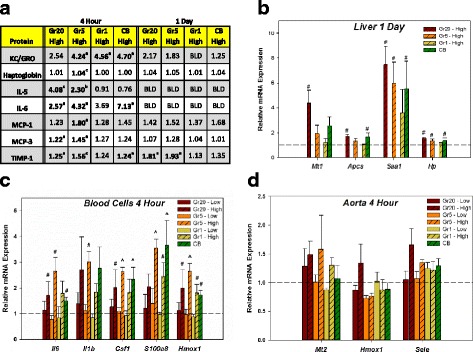
Serum Proteins and Systemic Gene Expression. Data in the table (**a**) represents fold-increase for serum proteins that were significantly different for one or more groups at 4 h and 1 day post-exposure based on RodentMap® v2.0; only high dose groups were analyzed. BLD – below the limit of detection; ^*a*^statistically different from DM only; ^*b*^statistically different DM, Gr1 - High and CB - High groups; ^*c*^statistically different from all groups; *p* < 0.05. **b** Relative mRNA expression by qPCR of select genes in liver at 1 day post-exposures. Low dose exposures were not evaluated in liver as pilot experiments showed no response. **c** Relative mRNA expression of select genes by qPCR in whole blood cells at 4 h post-exposure (**d**) Relative mRNA expression of select genes by qPCR in aorta at 4 h post-exposure. *significantly different from all groups; #significantly different from DM only; ^significantly different from DM, Gr20-Low, Gr5-Low, and Gr1-Low; *p* < 0.05

The same battery of proteins analyzed in BALF was analyzed in the serum at 4 h and 1 day post-exposure for DM and high dose groups including CB (Fig. 10a). In general, effects were greater in the Gr20 and Gr5 groups in comparison to Gr1. KC/GRO was the only protein found to be increased in serum collected from Gr1-exposed mice at 4 h post-exposure. KC/GRO, IL-6, and TIMP-1 were increased 4 h post-exposure in the CB exposed mice, corresponding to elevations in RNA levels at that time (Additional file 4: Table S3). The only mediator found to be increased in serum by 1 day post-exposure was TIMP-1 in the Gr20- and Gr5-exposed mice. No mediators were elevated in the Gr1 or CB groups at 1 day. Expression of liver acute phase genes, *Apcs, Saa1*, and *Hp* was assessed in the high dose groups only, and were found to be increased for the Gr20, Gr5, and CB groups (Fig. 10b). There was no change with Gr1 exposure at the high dose. Relative mRNA expression from whole blood cells was screened by the pre-designed array (Additional file 3: Table S2) and several genes were confirmed by qPCR (Fig. 10c). All genes analyzed by qPCR were significantly increased in the Gr5 high dose. High dose exposure to Gr20, Gr1, and CB resulted in at least 2 genes significantly increased in each group. There was a decrease in oxidant protective genes (*Sod2* -1.79; *Cat* -1.96; *Nqo1* -1.64 fold change) in the Gr5 group compared to DM in the analysis of the pre-designed array. No effects were seen in the low dose exposures. In the aorta, although there was a general trend for altered gene expression in the Gr20 high dose group, there were no significant increases in any of the genes selected for screening (Fig. 10d).

## Discussion

There is currently very little information available on airborne concentrations of graphene-based or graphite nanomaterials in occupational settings with the exception of one study by Lo et al. [19], that examined engineering controls for graphene platelets at a facility during manufacturing and handling processes where the highest concentrations were recorded at 2x10^6^ particles/cm^3^ during a post-treatment process. Based on the current lack of relevant workplace exposure data, doses for the present study were selected based on previous research that investigated toxicity of MWCNT Mitsui 7, a relatively toxic carbonaceous nanomaterial know to produce lung fibrosis [18, 20] and recently categorized by the International Agency for Research on Cancer (IARC) as a Group 2B (possibly carcinogenic to humans) [21, 22], for control purposes. A previous study showed that approximately 40 μg of MWCNT in the lung was equivalent to 76 years of a worker at the average facility exposure levels, and 4 μg was equivalent to 7.6 years, representing a more relevant worker exposure [18, 20]. While detailed exposure assessments have not been performed in facilities that handle graphene, the end-product applications have similarities to MWCNT and dry powder handling would be expected [23]. Therefore, the doses selected in this study were based on the MWCNT studies to attempt to reflect relevant workforce exposures (4 μg) and developing pathology (40 μg) as an early tier screen for general toxicity and potency classification of graphite nanoplates.

The goal of the current study was to characterize pulmonary and systemic toxicity associated with lung exposure to non-oxidized graphite nanoplates, a member of the graphene nanomaterial family, with respect to variation in lateral size of the material. To date, this is the first in vivo study to perform a size comparison of graphene nanomaterials of like composition. The materials investigated in these studies were identical in terms of structural and chemical composition, and zeta potential in the vehicle control (indicative of stability in solution and surface charge). The materials were also shown to be identical in terms of surface impurities that may contribute to reactivity as measured by ESR; however, in an acellular assay to determine reactivity by evidence of antioxidant depletion (Fig. 4), on a mass basis the order of reactivity followed as Gr1 > Gr5 > Gr20 ≥ CB. When normalizing the data to the surface are, it was found that reactivity was greatest for the Gr5 sample followed by the Gr20 sample, and was lowest for the Gr1 and CB which were comparable to each other. The materials also differed in lateral dimension, layer number (thickness), surface area, and degree of agglomeration in DM. All materials were found to agglomerate in DM, and the agglomerate size ranges varied in proportion to the lateral dimension.

Mice in the current studies were exposed to 4 or 40 μg of either Gr20 (~12 μm lateral x ~21 nm or 72 layers thick), Gr5 (~5 μm lateral x ~25 nm or 84 layers thick), Gr1 (~2 μm lateral x ~8 nm or 28 nm thick), or CB (~20 nm diameter) as a reference particle, in DM by a single pharyngeal aspiration. The flake or platelet-like shape presents a challenge in estimating deposition in the lung. Sanchez et al. [15] developed a model predicting regional deposition of plate-shaped materials with 0.5, 5, or 25 μm diameter in the lateral dimension relative to layer number, illustrating an alveolar deposition efficiency as high as 45 % for the small particle and up to 10 % for the largest particle. Additionally, Schinwald et al. [8] developed a calculation for the aerodynamic diameter of plate-like particles taking into account thickness, diameter, and densities, demonstrating that a particle with a lateral dimension of up to 30 μm would have an aerodynamic diameter of only 3.26 μm. These models collectively indicate that all three graphite nanoplate samples in the study presented here, Gr20, Gr5, and Gr1, would be considered respirable in humans.

The findings of this study showed that the systemic response following pulmonary exposure was transient. The greatest responses in liver and blood (cells and serum) were observed at 4 h and 1 day post-exposure (Fig. 10) in the high dose Gr20 and Gr5 groups, followed by the CB then Gr groups. These responses did not persist beyond the early time points post-exposure. Histopathological evaluation of the three graphite nanoplates showed no evidence of the development of fibrosis or granulomatous lung disease. The only histologic changes in the lung were related to injury and inflammation and were observed in the Gr20 and Gr5 groups scored as minimal. These responses either resolved over time or remained minimal at 2 months following exposure. No pulmonary inflammation was observed at the low dose exposures in any of the Gr groups, a dose that may prove to be more occupationally relevant based on findings from MWCNT facility studies discussed above.

The greatest changes were observed in the lung at the high doses of the larger sizes of graphite nanoplates in terms of inflammation, relative to that of Gr1. Increased inflammation was demonstrated as BAL granulocyte infiltration into the lung (neutrophils and eosinophils; Fig. 7b&c) and increased chemokines/cytokines in BAL fluid (Tables 2 and 3) following high dose exposure to the larger Gr20 and Gr5 particles beginning at 4 h post-exposure. The increase peaked at 1 day and persisted up to 1–2 months following exposure, with a trend for a decrease over time. Inflammation measured as neutrophil influx was present only at 1 day post-exposure in the high dose Gr1 and CB groups, and to the greatest degree in the CB group relative to all groups at this time point, agreeing with the largest induction of MIP-2 (Cxcl2) and KC (Cxcl1) (Table 2). No eosinophil influx was observed for the CB or Gr1 groups, corresponding with minimal changes in IL-5 and eotaxin in comparison to Gr5 and Gr20 (Table 2). Similar patterns in cytokine responses, whereby elevations persist for longer duration for the Gr5 and Gr20 groups, are also evident and are discussed below. These differential responses to particle exposure may be related to differences in the physico-chemical properties of the materials. In addition to the larger lateral dimensions and agglomerate structures of Gr5 and Gr20, these two particle were more reactive when normalized to surface area of the material. These characteristics may provide a possible explanation for inducing an earlier (4 h post-exposure) and more persistent response over time as compared to the high surface area materials of CB and Gr1, which caused cellular inflammation at only at day 1 post-exposure. Interestingly, the greatest degree of neutrophil influx at day 1 was caused by CB relative to the other Gr materials, including Gr1 which has twice the surface area as CB. Particle size in the acute response may also be playing a role in the strong neutrophil response as CB had the smallest primary particle size of all the materials. Further investigation is necessary to understand if this response could be related to the primary particle size in conjunction with high surface area. In addition, differences in patterns of inflammation and irritant response in relationship to particle properties such as reactivity also warrants further investigation.

Although the study was not designed to address mechanisms of toxicity related to specific cellular responses, several points are worth discussion related to other investigations of graphene-based nanomaterials. In recent years, formation of the NLRP3 inflammasome has been implicated as having a role in pathways to inflammatory diseases and fibrosis following particle or fiber exposure in the lung [24], including high aspect ratio nanoparticles such as MWCNT ([25–27]. Schinwald et al. [9] have also demonstrated the likely formation of the inflammasome following graphene exposure. This study did not directly assess inflammasome formation in relationship to the acute inflammatory responses, however, two proteins associated with the formation of the inflammasome were measured, IL-18 and IL-1β. An increase in IL-18 in BAL fluid was observed 1 day following exposure in all high dose groups, and IL-1β was variably increased among groups in BAL at this time point also. Gene expression analysis of IL-1β in lung tissue was also increased at 4 h for all Gr exposures and at 1 day for the Gr5 and Gr20 groups. As with most BAL protein parameters measured, the increased level of these proteins did not persist beyond 1 day, with the exception of an increase in IL-18 in the Gr5 group at 2 months. This study currently does not provide enough inflammasome-specific data to draw conclusions related to its likely absence over the majority of the time course and whether this correlates to the lack of fibrotic development in the lung following graphite nanoplate exposure.

A significant increase in eosinophil influx following exposure to the larger graphite nanoplates, Gr20 and Gr5, was observed, particularly at days 1 and 7 post-exposure, which resolved to a degree over time (Fig. 7c). The study design does not specifically address whether this is part of the overall inflammatory response, a specific irritancy response, or part of a greater immune response indicative of whether the material could act as a sensitizer or contribute to allergic responses in any way. Eotaxin, a protein involved in eosinophil recruitment in inflammation and allergic response, was significantly elevated in all groups with Gr5 > Gr20 > CB > Gr1. In terms of T cell-related cytokines that are implicated in airway disease, gene expression of IL-5, Il-13, and IL10 were elevated at 4 h post-exposure in Gr-exposed groups, and IL-5 expression remained elevated at 1 day post-exposure in the Gr5 and Gr20 groups. IL-5 was also elevated 1 day post-exposure in the BAL to a greater degree in Gr5 and Gr20 when compared to all groups; however the elevation was transient. Shvedova et al. has shown that exposure to another form of graphene material, graphene oxide, can alter airway hyperresponsiveness in a murine model of asthma [11], however, in that particular study the Th2 cytokines (IL-4, IL-5, and IL-13) were reduced. Further investigation is needed to determine the physico-chemical characteristics of graphene nanomaterials that may be involved in airway disease or lack thereof.

In addition to increased inflammatory and immune markers in the BAL fluid of Gr20- and Gr5-exposed mice, gene expression in tissue followed a similar pattern as BAL protein analysis, with increased mRNA expression of genes encoding pro-inflammatory factors, IL-6 (*Il6*), macrophage inflammatory protein (MIP)-2 (*Ccl2*), monocyte chemotactic protein (MCP)-1 (*Cxcl2*) and macrophage-derived chemokine (MDC) (*Ccl22*) (Fig. 9). The expression of these genes persisted longest in the Gr20 and Gr5 groups, but diminished in all groups over time. BAL fluid markers associated with tissue remodeling (MMP-9, TIMP-1) were also increased to a greater degree at day 1 post-exposure and persisted longer when compared to Gr1- and CB-exposed mice. Interestingly, gene expression of osteopontin, a potential marker of the development of lung diseases including fibrosis and cancer, had begun to increase by 1 and 2 months post-exposure in the Gr20 and Gr5 high dose groups; however, no histopathological markers of disease were present at 2 months and protein levels of MMP-9 and TIMP-2, although elevated when compared to the DM group, were decreasing over time. Despite the ~50 % particle clearance at 2 months, this finding warrants examination of clearance and effects at time points later than 2 months following lung exposure to determine if the trend continues and fibrotic changes occur later in recovery.

The characterization and toxicity data taken together suggest that the pattern of toxicity for the Gr5 and Gr20 samples may have been driven by the surface reactivity per unit surface area and/or the overall size of the material (lateral size layer number and possibly agglomeration), rather than surface area (measured in the powder form) or particle number, where Gr5 and Gr20 were lower in both parameters when compared to Gr1 and CB. Although a large surface area is a known driving force in toxicity of nanomaterials, this factor may be more critical for the early acute responses; whereas with high-aspect ratio materials such as graphene, shape, reactivity, dissolution rate (biopersistence), and durability may be more critical [28–31]. The agglomerate size for the larger particles raises the question of whether frustrated phagocytosis could be playing a role in toxicity of these high aspect ratio materials. However, in this study, there was no evidence of granuloma formation in Gr5 or Gr20-exposed mice, and multinucleated macrophages, although present, were found infrequently in the BAL (Additional file 1: Figure S1), suggesting if this was occurring it was present at a very low level. More research is necessary to clearly define the role of the physico-chemical properties in toxicity as well as the mechanisms of action occurring at the cellular and molecular level.

In general, the data presented here, relative to graphene-based nanomaterials that have not gone through an oxidation process, are in agreement with other studies whereby pulmonary exposure causes a degree of inflammation only at the early time points after exposure with resolution over time [6, 8–10]. As mentioned earlier, terminology for these materials is variable among investigators, and for the non-oxidized forms of graphene, the studies have referred to the materials as pristine graphene, graphene nanosheets/nanoplates/nanoplatelets, or graphite nanosheets/nanoplates/nanoplatelets. Shin et al. [10] found no pulmonary toxicity associated with an estimated deposited dose of 102 or 18 μg of graphene (~0.55 μm laterally x 8 nm thickness, referred to as graphene) in the lungs of rats at any time point up to 28 days following a 5-day dose-response repeated inhalation study in rats, a dose comparable to the amount of graphite nanoplates deposited in the current study. The studies by Schinwald et al. [8, 9] examined pulmonary effects following exposure to 50 μg of graphene in mice (referred to as graphene nanoplatelets [9] and then as pristine graphene [8]). In addition to having a comparable dose to the high dose in the current study, the graphene particle used by Schinwald et al. was similar in size (~5 μm laterally x 100 nm thick; ~100 m^2^/g surface area) to the Gr5 primary particle dimensions in the current study. Graphene was found to induce inflammation and injury at 1 day following aspiration, with granulomatous lesions in the bronchiole lumen and alveolar region, as well as pleural inflammation following pleural injection [9]. Responses were followed up to 6 weeks post-exposure [8] and were found to show resolution over time with no development of fibrosis or increase in collagen, which is in agreement with present study.

Further, Ma-Hock et al. [6] conducted a dose response comparative inhalation study of MWCNT, graphene, graphite nanoplatelets, and low surface area CB. No toxicity was observed following 5 days (6 h/day) inhalation of the highest tested dose, 10 mg/m^3^ (~200 μg deposited in the lung) of large graphite nanoplatelets (up to 30 μm diameter of primary particle and > 50 μm diameter agglomerated; mass median aerodynamic diameter of 2.7/3.4 μm). Interestingly, in the study by Ma-Hock et al. [6] the 5 day exposure to graphene (primary particle up to 10 μm lateral size and ~40 μm agglomerate size; mass median aerodynamic diameter < 0.4 μm) at a dose of 2.5 mg/m^3^ for 6 h/day, with comparable deposition to the present study, produced a low (relative to MWCNT exposure) but significant effect up to 1 month post-exposure with increased inflammatory mediators in lavage and lung tissue, including osteopontin. In the present study, there was also an increase in osteopontin in lung tissue at 1 and 2 months post-exposure to the larger Gr5 and Gr20 particles, although no pathology in tissue was observed with the graphite nanoplates. However, in the study by Ma-Hock et al. [6], at 10 mg/m^3^ of graphene, there was a greater significant increase in inflammation and formation of microgranulomas in the lung that persisted up to 1 month post-exposure. The findings suggest that pristine graphene was more potent than graphite nanoplates, which produced no effect at the 10 mg/m^3^ dose at comparable mass doses, but is far less potent than MWCNT which caused significant toxicity at one fourth the dose. The authors concluded that potency did not correlate well with lung burden in terms of mass, volume, or exposed surface, where the larger aerodynamic diameter particle (MWCNT) had the greatest toxicity; and that reactivity or a combination of properties may be the driving factor in potency. Although toxicity was far less for the materials in the present studythe material with the larger aerodynamic diameters (Gr5 and Gr20) and greater reactivity per m^2^ were more toxic, suggesting size and/or reactivity as possible factors.

Other studies of graphene nanoplate materials have found adverse effects following pulmonary exposure [4, 7]. Park et al. [7] examined lung responses in mice following intratracheal administration of a graphene particle similar in lateral size and surface area of Gr1 at doses of 2.5 and 5.0 mg/kg in mice, referred to as pristine graphene. Differences in the current study versus the findings in Park et al. [7] may be attributable to dose, where the highest dose in the present study of 40 μg per mouse was equivalent to only 2.0 mg/kg, and possibly to differences in the graphitic nature and defects present in the particles, where the particle used by Park et al. [7] had fewer layers and may have been lower in defects and disorder in structure. In a study by Duch et al. [4], mice were treated by aspiration with differing graphene materials referred to as dispersed pristine graphene (in 2 % Pluronic F 108NF®), aggregated pristine graphene, or graphene oxide flakes, at 50 μg per mouse, a dose comparable to that of the current study. Aggregated pristine graphene was generally less toxic than graphene oxide, but more toxic than dispersed pristine graphene at 1 day post-exposure. At 21 days post-exposure, histology showed that only the aggregated material caused what was described as a “patchy” fibrosis, although there were no significant increases in collagen as determined by Sirius red staining in any of the treatments. A more severe, persistent inflammation was evident in lungs of mice exposed to graphene oxide. Particles used in this study were small in both lateral dimension (~200–500 nm) and thickness (1.2–5 nm) when compared to the Gr20, Gr5, and Gr1 in the present study. Interestingly, the aggregated material, which would presumably have a greater lateral dimension, or aerodynamic diameter, within a given aggregate, produced a greater effect than the dispersed material, suggesting a role of agglomerate size in toxicity, which is similar to the possible size-dependent effects observed in the current study.

The studies discussed above suggest that the size, graphitic nature/purity, and possible related reactivity are important factors for pulmonary toxicity and support the findings of the present study that shows that size/agglomeration and/or reactivity are factors in toxicity. The current study, however, only examined non-oxidized materials, and the toxicity that resulted showed resolution over time. It is well understood that composition plays a critical role in toxicity. In general, in vivo studies have demonstrated that the oxidized forms of graphene nanomaterials cause a greater pulmonary toxicity than that of what was referred to as pristine graphene or graphite nanoplates/nanoplatelets [4, 5, 11]. As discussed above, Duch et al. [4] found graphene oxide to be more toxic than the pristine form of graphene. Li et al. [5] demonstrated that respiratory exposure to graphene oxide in mice resulted in a dose-dependent increase in lung toxicity and development of fibrosis at 3 months post-exposure to a dose of 10 mg/kg. Graphene oxide has also been shown to cause airway remodeling and hyperresponsiveness in an OVA-induced asthma model in mice when administered prior to the sensitization phase of the model [11]. The same study also determined that graphene oxide caused a decrease in Th2 cell responses in the asthma model. In vivo data showing increased toxicity of oxidized graphene nanomaterials are further substantiated by numerous in vitro studies in a host of different cell types as reviewed in detail by Seabra et al. [32] and Bianco [33]. Studies are underway by our laboratories to examine pulmonary and systemic toxicity due to variations in oxidation state along the life-cycle of a given graphene nanomaterial.

## Conclusions

In conclusion, the findings of the present study suggest that pulmonary exposure to graphite nanoplates of similar composition but with larger lateral dimensionsand increased surface reactivity per unit surface area causedinjury and inflammation in the lung as early as 4 h post-exposure. The increase peaked at 1 day and persisted up to 1–2 months following exposure, with a trend for a decrease over time The pattern of inflammation differed from that of the smaller graphite nanoplate sample and from carbon black nanoparticles, both of which were higher in surface area when compared to the larger materials. These findings suggest a role for surface properties and/or lateral particle size, and possibly agglomerate size, in initiating toxicity. The pulmonary and systemic effects observed early after exposure did show resolution over time for all groups. Taken together with results of studies on other graphene-based nanomaterials, toxicity of non-oxidized forms of graphenes may depend on size and surface reactivity, as well as agglomeration of materials, and are likely less toxic than various forms of graphene oxides, for which toxicity has been shown to be more persistent with less resolution over time, and may be dependent on a different set of physicochemical characteristics including surface chemistry as well as reactivity.

## Methods

### Particle characterization

#### Particle size

Three different sizes of graphite nanoplates (Gr) were provided by Cabot Corp., Billerica, MA. The samples were manufactured to have primary particle dimensions of 20 μm lateral (Gr20), 5 μm lateral (Gr5), and < 2 μm lateral (Gr1). Carbon black (CB), ~15 nm diameter, was purchased commercially (Printex 90, Degussa- Heuls, Germany) to serve as a particle control. FESEM (Hitachi Model S-4800, Japan) was used to visualize and verify size of the particles in the dry powder form.

#### Surface area

Surface area was measured on the powder form of the materials by gas adsorption using a Quantachrome NOVA 2200e surface area analyzer and ultra-high purity nitrogen adsorbate. Specific surface area was determined by using the multipoint (7 recorded points) Brunauer, Emmett, and Teller (BET) method for relative pressures between 0.05 and 0.35 [34].

#### Structural analysis

XRD was performed to determine the crystalline phase of graphite present, as well as the number of layers present in each sample. Room temperature powder x-ray diffraction (XRD) patterns were acquired with a Rigaku diffractometer (D/Max B) equipped with CuKα source (λ = 1.54185 Å) and the d-spacings and 2ϴ positions of the observed lines were compared to published reference patterns. Typically, scans covered the 2ϴ range of 5° to 70° and were collected at a step size of 0.06° with a counting time of 5 s at each step. Interpretation of the XRD patterns was based on the basic Bragg’s law for diffraction: 2d sinϴ = n*λ*, where d is the separation of the (hkl) planes responsible for the particular (hkl) Bragg peak, ϴ is the Bragg angle of a peak, n is the order of diffraction (1, 2, 3, etc.), and *λ* = 1.54185 Å is the Cu K_α_ wavelength of the x-rays used in these investigations. The following information about the graphite structure was extracted from the XRD patterns: form (2H or 3R), degree of graphitization, crystallite size, and number of layers. The lattice parameters of 2H graphite are: c = 6.708 Å, a = 2.4614 Å, c = 2 d_(002)_ and the degree of graphitization $\overline{\mathit{\mathsf{g}}}$ is defined as [35, 36].

1 $$ \overline{\mathit{\mathsf{g}}}=\frac{3.440-d(002)}{3.440-3.354} $$

The apparent crystallite size along the c- (L_c_) and a- (L_a_) directions are given by [35, 36].

2 $$ {\mathrm{L}}_{\mathrm{c}}=\frac{(0.91)\lambda }{\beta cos\theta}, $$

and

3 $$ {\mathrm{L}}_{\mathrm{a}}\frac{(1.84)\lambda }{\beta cos\theta} $$

where

*β* is the instrument-corrected full-width at half maximum (FWHM) of the (002) line near 2ϴ ≈ 26° in radians for L_C_; the FWHM for the (101) or (112) in radians are used for determining L_a_. By expressing Bragg’s law in terms of *λ*, equation 2 for L_C_ can be used to derive the number of graphene layers along the c-direction.

4 $$ \#\mathrm{layers}\ {\mathrm{N}}_{\mathrm{c}}=\frac{\mathrm{Lc}}{d(002)}=1.8\  \tan \uptheta /\beta . $$

#### Particle preparation in DM

For in vivo studies, aspiration requires the suspension of the particles in a physiologically compatible vehicle for delivery to the animal. Gr and CB samples were suspended in a well-characterized DM (0.6 mg/ml mouse serum albumin + 0.01 mg/ml dipalmitoyl phosphotidyl choline in phosphate buffered saline [14]) designed to mimic the fluid lining of the lung at a concentration of 0.8 mg/ml, equivalent to the high dose (40 μg particle/50 μl DM) for in vivo studies. The samples were then sonicated at 10 W for 5 s each with a probe sonicator. The low dose (4 μg/50 μl) of Gr was prepared by diluting the high dose 1:10 in DM. Particle preparations were made on the day of use for both characterization and in vivo studies.

#### Agglomerate size distribution in DM

Light micrographs of the high dose aspiration preparations (0.8 mg/ml DM) of Gr20, Gr5, Gr1, and CB were acquired (60 and 100x magnification). Corresponding agglomerate/particle diameter size measures made from point count measurements on the light microscope (*n* = 214–491 measures per sample) were acquired and the mean agglomerate size, measured from the greatest distance across agglomerates, and distribution were determined. DLS (Microtrac Inc., San Diego, CA) was used to verify the size range for the smaller particle samples, Gr1 and CB. Gr20 and Gr5 could not be quantified with DLS due to size and density of agglomerates.

#### Zeta potential in DM

Zeta potential was determined by electrophoretic mobility. For each material, a stock suspension (1 mg/mL) prepared in DM was subjected to ultrasonic agitation using a 3 mm probe tip for 5 min (delivered energy = 1650 J) and further diluted using fresh DM. The DM had pH 7.4, as determined using a calibrated electrode attached to a volt meter. The parameters for the dispersant were based on PBS, the major constituent of DM (refractive index = 1.334, viscosity = 0.9110 cP, dielectric constant = 79.0, and Smoluchowski approximation, f(ka) value = 1.5). All measurements were performed at 25 °C using a Malvern Zetasizer Nano ZS90 (Worcestershire, UK) equipped with a 633 nm laser at a 90° scattering angle. Samples were equilibrated inside the instrument for two minutes, and five measurements, each consisting of five runs, were recorded.

#### Surface reactivity

Generation of hydroxyl radicals on the particle surface as an indicator of surface impurities implying reactivity was evaluated by ESR using an EMX spectrometer (Bruker Instruments Inc., Billerica, MA) and a flat cell assembly. This technique involved the addition-type reaction of a short-lived radical with a diamagnetic compound (spin trap) to form a relatively long-lived free radical product (the spin adduct), which can be observed by conventional ESR [37]. For this study, hydroxyl radical was generated from a Fenton-like reaction system after the Gr or CB samples were suspended in dispersion media (at a dose equivalent to the high dose used in this study) and were combined with H_2_O_2_ [1 mM] in the presence of 100 mM of the spin trap 5,5-dimethyl-1-pyrroline N-oxide (DMPO) and PBS to a final volume of 1 ml. Reactions were allowed to incubate 3 min at room temperature before measurement and then transferred to a flat cell for ESR measurement. Signal intensity of the spin adduct, which corresponds to the amount of a given radical species, was determined by integration of the characteristic wave form for that radical. The wave form was then measured and quantified and compared to a positive control at the same concentration (coal containing α-quartz silica).

To detect the free radicals generated by particles, a previously reported method utilizing DCFH was employed [38, 39]. Briefly, DCFH-diacetate was chemically hydrolyzed to generate DCFH using 0.01 N NaOH for 30 min at room temperature. This solution was further neutralized using 0.1 M PBS. Nanoparticle suspensions were prepared in DM as above. To evaluate a dose response, a reaction mixture containing a final concentration of 10 μM DCFH (with and without 0.1 unit/ml horse radish peroxidase) was added to wells containing nanoparticles at 6.25, 12.5, 25 and 50 μg/ml. All experiments were conducted in triplicate with water, DM and H_2_O_2_ as controls. The kinetics of DCFH oxidation was monitored for 60 min with readings every 10 min at 485 nm excitation and 530 nm emission. All measurements were conducted with under low light exposure.

A third cell free assay system was used based on antioxidant (DTT, dithiothreitol) depletion to determine the oxidative potential of the materials used in the current study. This method has been used to determine the oxidation potential of carbon-based and metal oxide nanoparticles by several groups [40–43]. The oxidative capacity of the nanomaterials was determined by its ability to consume DTT. The DTT content left in the reaction mixture was determined by reacting it with 5,5′-dithio-bis-(2-nitrobenzoic acid) (DTNB). Reaction with DTNB is rapid and stoichiometric resulting in formation of 2-nitro-5-thiobenzoate (TNB), a colored by product that can be calorimetrically detected by measuring absorption at 412 nm. Nanomaterials suspensions were prepared in DM as described above. The reaction was conducted with samples in triplicates with PBS and DM as controls in a 96-well black plate with optical bottom. The microplate was shielded from light and was placed under constant agitation. 25 μl of 50 μg/ml nanoparticle suspension was reacted with 25 μl of 100 μM DTT. After 30 min of reaction, the reaction was quenched using 50 μl of 10 % trichloroacetic acid. This reaction mixture was further neutralized by addition of 180 μl of Tris-HCl buffer pH 8.9. The DTT left in the reaction mixture was determined by exposing it to 5 μl of 10 mM DTNB in water and calorimetrically determined by absorption at 412 nm. As numerous studies have shown that surface area is an important dose metric for nanoparticle-related toxicity, in addition to plotting the DTT consumption based on nanoparticle mass (nM/μg), we have also plotted the DTT consumption based on surface area (nM/M^2^) calculated through BET.

### Animals

Specific pathogen-free, male C57BL/6 J mice, ~8 weeks of age, were obtained from Jackson Laboratory (Bar Harbor, ME) for use in this study. All mice were individually housed in polycarbonate ventilated cages with HEPA-filtered air in the Association for Assessment and Accreditation of Laboratory Animal Care (AAALAC) -approved National Institute for Occupational Safety and Health (NIOSH) Animal Facility, and provided food [Harlan Teklad Rodent Diet 7913 (Indianapolis, IN)] and tap water ad libitum in a controlled humidity and temperature environment with a 12 h light/dark cycle. Animals were allowed to acclimate for 1 week in the facility prior to use in the study. All procedures in the study comply with the ethical standards set forth by the Animal Welfare Act (enforced by the United States Department of Agriculture) and the Office of Laboratory Animal Welfare (OLAW). The studies were approved by the NIOSH Health Effects Laboratory Division (HELD) Institutional Animal Care and Use Committee within the Center for Disease Control (Public Health Services Assurance Number A4367-01) in accordance with an approved institutional animal protocol (protocol number 12-JR-M-001).

### In vivo exposure and study design

To evaluate pulmonary and extrapulmonary toxicity following lung exposure to a well-characterized group of graphene nanomaterials, a dose-response time course study in mice was conducted. On day 0, two sets of male C57BL/6 J mice were exposed by pharyngeal aspiration to 4 or 40 μg of Gr20, Gr5, or Gr1 in DM, DM alone (vehicle control), or 40 μg of CB (particle control). Mice were fully anesthetized with isoflurane, placed on a slanted board and suspended by the incisors. The mouth was opened and tongue moved aside, while a 50 μl aliquot of sample was pipetted to the base of the tongue. The animal was restrained until two full breaths were completed (but not longer than 15 s), then placed on its side and monitored for recovery from anesthesia. Animal exposures were conducted utilizing a randomized block design (*n* = 8 per group per time point with the exception of the 4 h time point where the group size was *n* = 6). Mice were humanely euthanized with an overdose of sodium pentobarbital (100–300 mg/kg bwt; Sleepaway; Fort Dodge Animal Health; Madison, NJ) at 4 h, 1 day, 7 days, 1 month, and 2 months post-exposure. The lungs of one set of mice underwent BAL (*n* = 6–8 per group per time point) to collect fluid and cells for pulmonary toxicological analyses. In the second set of mice (*n* = 6–8 per group per time point), the left lung was ligated and frozen in liquid nitrogen for RNA analysis while the right lungs were fixed, sectioned, and stained for histopathology and morphometric analysis of deposition and clearance. Blood, heart, aorta, and liver were collected to evaluate cardiovascular parameters of toxicity. Tissues for RNA isolation were snap-frozen in RNA-free tubes in liquid nitrogen at the time of tissue collection.

### Pulmonary deposition/clearance and histopathology

The right lungs of mice were pressure fixed in 10 % neutral-buffered formalin, paraffin embedded, and sectioned for histopathological and morphometric analyses. Tissue sections from right lungs of control mice and mice treated with the high doses of Gr20, Gr5, Gr1, and CB were stained with Sirius red and hematoxylin. The distribution of particle at 4 h post-exposure in the lungs was determined by counting the occurrence of Gr or CB under an eyepiece point counting overlay using standard morphometric point counting methods [44]. Point counting categories were subdivided into points over Gr or CB in airway or points over Gr or CB in the alveolar region. Airway regions were defined as those containing airway tissue (airway epithelial cells-basement membrane and tissues of the broncho-vascular cuff), airway lumen, and associated blood vessels greater than 25 μm. Alveolar regions were those containing alveolar tissue, alveolar macrophages, and alveolar air space. To accomplish the counting, the optical system for enhanced darkfield microscopy was employed that consisted of high signal-to-noise, darkfield-based illumination optics adapted to an Olympus BX-41 microscope (CytoViva, Auburn, AL 36830), in combination with light microscopy. The use of this microscope enables evaluation of nanomaterials as small as 10 nm, allowing for detection of the smaller agglomerates described in this study [45, 46]. An eyepiece counting overlay consisting of 11 by 11 lines (121 total points for each throw of the overlay) was used with a 100x oil immersion objective. A grid pattern for throws of the counting overlay was used in order to insure a uniform sampling of the section, which did not overweight interior points. The counting overlay throws of the eyepiece were positioned over the section at 12 uniformly spaced grid points in both X and Y co-ordinates. These 12 grid points were determined using the stage micrometer scale to measure the X and Y bounds of the section. Using the bounding rectangle of these co-ordinates a 3 by 4 grid was selected and the 12 intersections were used as the center point for each of the eyepiece counting overlay throws. For each animal, three sections were counted, and the counts for the airway and alveolar region were summed. Each counting category was divided by this total and multiplied by 100 to express the results as a percentage of total lung burden. Similar analyses were performed at 7 days and 2 months post-exposure to evaluate clearance of the material from the lungs in total and by region.

For histopathological analysis, two sections of right lung tissue from each animal in each treatment group, both low and high doses, as well as controls were evaluated: one section was stained with hematoxylin and eosin (H&E) for parameters of injury and inflammation, and another section was trichrome-stained for analysis of fibrosis (*n* = 6 per group per time point). Slides were quantitatively analyzed by a certified veterinary pathologist at Charles River Laboratories (Frederick, MD), who was blinded to the treatment groups. Indices of inflammation, injury, and fibrosis were scored on scale of 0–5, where 0 = no observed effect, 1 = minimal response, 2 = mild response, 4 = moderate response, and 5 = severe response.

### BAL Cellular and Fluid (BALF) analysis

In a separate group of mice, BAL was performed on the lung in order to obtain pulmonary cells and fluid for analysis of indicators of lung injury, inflammation, and cellular activity (*n* = 8 per group per time point). Following euthanasia, the trachea was cannulated, the chest cavity was opened, and BAL was performed on the whole lungs. The acellular fraction of the first lavage was obtained by filling the lung with 0.6 ml PBS, massaging for 30 s and withdrawing. This concentrated aliquot was retained, kept separately, and was designated as the first fraction of BALF. The following aliquots were 0.6 ml in volume, instilled once with light massaging, withdrawn, and combined until a 2.4 ml volume was obtained. For each animal, both lavage fractions were centrifuged (10 min, 1600 rpm), the cell pellets were combined and resuspended in 1 ml of PBS for cell counts and differentials, and the acellular fluid from the first fraction was retained for analysis of LDH activity and protein content.

The total numbers of BAL cells collected from mice were counted using a Coulter Multisizer II (Coulter Electronics; Hialeah, FL). Cell differentials were performed to determine the total number of alveolar macrophages (AM), neutrophils, lymphocytes, and eosinophils. Briefly, 10^5^ cells from each mouse were spun down onto slides with a Cytospin 3 centrifuge (Shandon Life Sciences International; Cheshire, England) and labeled with Leukostat stain (Fisher Scientific; Pittsburgh, PA) to differentiate cell types. Two hundred cells per slide were counted, and the percentages of AM, neutrophils, lymphocytes, and eosinophils were multiplied by the total number of cells to calculate the total number of each cell type. In addition, images of the cytospin were collected at 40x magnification to illustrate particle burden in macrophages at various time points post-exposure.

Measurements of LDH activity in BALF was obtained using a Cobas Mira analyzer (Roche Diagnostic Systems; Montclair, IN). LDH activity was quantified by detection of the oxidation of lactate coupled to the reduction of NAD^+^ at 340 nm. A battery of 60 proteins in BALF was measured using RodentMap® v2.0 for the 1 day, 7 days, and 1 month post-exposure time points and RodentMap® v3.0 for the 2 month time point (Myriad RBM, Austin, TX) (*n* = 4–6 per group per time point analyzed).

### RNA isolation and relative mRNA expression via qPCR

RNA was isolated from frozen lung and liver using the RNeasy Mini Kit (Qiagen, Valencia, CA, USA) following homogenization using a Tissue Lyser II (Qiagen, Valencia, CA). The aorta, following homogenization with the Tissue Lyser II, was isolated using the RNeasy fibrous tissue mini kit (Qiagen, Valencia, CA). Whole blood RNA was isolated using the Mouse RiboPure™-Blood RNA Isolation Kit (Ambion, Austin, TX) according to manufacturer’s directions and globin RNA was removed using the GLOBINclearTM kit (Ambion, Austin, TX). A 2 μl aliquot of each RNA sample was quantified using a NanoDrop ND-1000 spectrophotometer (NanoDrop Technologies, Inc., Wilmington, DE). Evaluation of gene expression was determined by standard 96-well technology using the StepOne™ (Applied Biosystems, Carlsbad, CA, USA) with pre-designed Assays-on-Demand™ TaqMan® probes and primers. Assessed genes for the lung included IL-6 (*Il6*, Mm00446190_m1), MIP-2 (*Ccl2*, Mm00441242_m1), MCP-1 (*Cxcl2*, Mm00436450_m1), MDC (*Ccl22*, Mm00436439_m1), and osteopontin (*Spp1*, Mm00436767_m1); liver genes included haptoglobin (*Hp*, Mm0516884_m1), metallothionein 1 (*Mt1*, Mm00496660_g1), serum amyloid A1 (*Saa1*, Mm00656927_g1), and serum amyloid P (*Apcs*, Mm00488099_g1); genes for whole blood cells included IL-6 (*Il6*, Mm00446190_m1) Il-1β (Mm00434228_m1), CSF-1 (Mm00432686_m1), S100a8 (Mm00496696_g1), heme oxygenase-1 (Hmox1 Mm00516005_m1); and genes for aorta included metallothionein 2 (*Mt2*, Mm00809556_s1, heme oxygenase-1 (*Hmox1*, Mm00516005_m1), E-selectin (*Sele*, Mm00441278_m1) Using 96 well plates, one μg of total RNA was reverse transcribed using random hexamers (Applied Biosystems) and Superscript III (Invitrogen, Carlsbad, CA). Nine μl of cDNA (1/10) was then used for gene expression determination. Hypoxanthine-guanine phosphoribosyltransferase was used as an internal reference for all tissues and 18S for whole blood cells. Relative gene expression was calculated using the comparative threshold method (2^-ΔΔCt^) with vehicle-treated mice serving as the reference group [47].

### Relative mRNA expression via TaqMan® arrays

An initial screen utilizing pre-designed TaqMan® arrays that included 93 target genes and 3 housekeeping genes was done for dispersion media, Gr1 high, Gr5 high, and Gr20 high (*n* = 3 or 4 per group). Lung (4 h and 1 day), liver (1 day), and whole blood cells (4 h) were screened for alterations in gene expression. The complete list of genes is included in the supplemental section (Additional file 3: Table S2). Samples were run according to the Applied Biosystems TaqMan® array user bulletin using the ABI 7900 (Applied Biosystems). A total of 1000 ng of total RNA converted to cDNA in a final volume of 200 μl (96a format) was used. The housekeeping gene contained within the LDA used for normalization was hypoxanthine-guanine phosphoribosyltransferase (HPRT). Relative gene expression was calculated using the comparative threshold method (2^-ΔΔCt^) with vehicle treated mice serving as the reference group.

### Serum protein analysis

Proteins quantified in serum collected 4 h and 1 day post-exposure were determined by Myriad/Rules Based Medicine (Austin, TX) by multiplex immunoassay RodentMap® v2.0.

### Statistics

Results for BAL analyses were expressed as means + standard errors and an analysis of variance (ANOVA) was performed to determine significant difference among treatment groups. Data for BAL protein parameter analyses were log transformed before testing for significance. Significant differences among groups were assessed by the Student-Newman-Keuls method. For morphometric studies, Bartlett’s test was used to test for homogeneity of variances between groups. When significant F values were obtained, individual means were compared to control using Duncan’s multiple comparison procedure. Because data from histopathology studies were inherently categorical, a nonparametric analysis of variance was performed using SAS, Inc. statistical programs using the Wilcoxon rank sum test. For all analyses, significance was set at *p* < 0.05. Data from Taqman® arrays and qPCR were log transformed and analyzed by one-way ANOVA generating a least squares mean table by Student’s *t*-test using JMP® Statistical Discovery Software. Differences were considered statistically significant at *p* < 0.05.

## Abbreviations

AAALAC, Association for Assessment and Accreditation of Laboratory Animal Care, AII, alveolar interstitial inflammation; AM, alveolar macrophages; ANOVA, analysis of variance; BAL, bronchoalveolar lavage; BALF, bronchoalveolar lavage fluid; BALT, bronchus-associated lymphoid tissue; BET, Brunauer, Emmett, and Teller; BHH, bronchiolar hypertrophy and hyperplasia; CB, carbon black; CNT, carbon nanotubes; DCFH, 2′–7′-dichlorofluorescin; DLS, dynamic light scattering; DM, dispersion media; DMPO, dimethyl-1-pyrroline N-oxide; DTNB, 5,5′-dithio-bis-(2-nitrobenzoic acid); DTT, dithiothreitol; ENM, engineered nanomaterial; ESR, electron spin resonance; FESEM, field emission scanning electron microscopy; FWHM, full width half maximum; Gr, graphite nanoplates; HRP, horseradish peroxidase; IARC, International Agency for Cancer Research; IL, interleukin; KC/GRO, growth-regulated alpha protein; LDH, lactate dehydrogenase; MCP, monocyte chemotactic protein; MDC, macrophage-derived chemokine; MIP, macrophage inflammatory protein; MMP, matrix metalloproteinases; MPO, myeloperoxidase; mRNA, messenger ribonucleic acid; MWCNT, multi-walled carbon nanotubes; NIOSH, National Institute for Occupational Safety and Health; OVA, ovalbumin; PBS, phosphate buffered saline; PCR, polymerase chain reaction; qPCR, quantitative polymerase chain reaction; RNA, ribonucleic acid; SAR, structure activity relationship; TBI, terminal bronchiolar inflammation; TIMP, tissue inhibitor of metalloproteinase; TNB, 2-nitro-5-thiobenzoate; XRD, x-ray diffraction.

## Additional files

Additional file 1: Figure S1. Images of cells recovered by BAL from animals exposed to DM or 40 μg of Gr20, Gr5, Gr1, or CB, at 4 h, 7 days, and 2 months post-exposure illustrating particle burden in macrophages from the alveolar region. (PDF 1663 kb)

Additional file 2: Table S1. List of total proteins analyzed by RodentMAP® analyses, abbreviations, and alternate nomenclature. (PDF 32 kb)

Additional file 3: Table S2. Genes contained in the pre-designed TaqMan® array. (PDF 22 kb)

Additional file 4: Table S3. Differentially expressed genes in lung tissue following exposure to 40 μg of Gr20, Gr5, or Gr1. (PDF 38 kb)

## References

[1] Novoselov KS, Geim AK, Morozov SV, Jiang D, Zhang Y, Dubonos SV, Grigorieva IV, Firsov AA (2004). Electric field in atomically thin carbon films. Science.

[2] Markets F (2015). The global market for graphene forecast from 2010 to 2025: production volumes, prices, future projections, and end user markets.

[3] Geim AK, Novoselov KS (2007). The rise of graphene. Nat Mater.

[4] Duch MC, Budinger GRS, Liang YT, Soberanes S, Urich D, Chiarella SE, Campochiaro LA, Gonzalez A, Chandel NS, Hersam MC, Mutlu GM (2011). Minimizing oxidation and stable nanoscale dispersion improves the biocompatibility of graphene in the lung. Nano Lett.

[5] Li B, Yang J, Huang Q, Zhang Y, Peng C, Zhang Y, He Y, Shi J, Li W, Hu J, Fan C. Biodistribution and pulmonary toxicity of intratracheally instilled graphene oxide in mice. NPG Asia Mat. 2013;5:e44. doi:10.1038/am.2013.7.

[6] Ma-Hock L, Strauss V, Treumann S, Küttler K, Wohlleben W, Hofmann T, Gröters S, Wiench K, van Ravenzwaay B, Landsiedel R. Comparative inhalation toxicity of multi-wall carbon nanotubes, graphene, graphite nanoplatelets and low surface carbon black. Part Fibre Toxicol. 2013;10:23. 10.1186/1743-8977-10-23 PMC3720229 23773277

[7] Park EJ, Lee GH, Han BS, Lee BS, Lee S, Cho MH, Kim JH, Kim DW. Toxic response of graphene nanoplatelets in vivo and in vitro. Arch Toxicol. 2014;89:1557-68. 10.1007/s00204-014-1303-x 24980260

[8] Schinwald A, Murphy F, Askounis A, Koutsos V, Sefiane K, Donaldson K, Campbell CJ (2014). Minimal oxidation and inflammogenicity of pristine graphene with residence in the lung. Nanotoxicology.

[9] Schinwald A, Murphy FA, Jones A, MacNee W, Donaldson K (2012). Graphene-based nanoplatelets: a new risk to the respiratory system as a consequence of their unusual aerodynamic properties. ACS Nano.

[10] Shin JH, Han SG, Kim JK, Kim BW, Hwang JH, Lee JS, Lee JH, Baek JE, Kim TG, Kim KS, et al. 5-Day repeated inhalation and 28-day post-exposure study of graphene. Nanotoxicology. 2015;9:1023-31. 10.3109/17435390.2014.998306 25697182

[11] Shurin MR, Yanamala N, Kisin ER, Tkach AV, Shurin GV, Murray AR, Leonard HD, Reynolds JS, Gutkin DW, Star A (2014). Graphene oxide attenuates Th2-type immune responses, but augments airway remodeling and hyperresponsiveness in a murine model of asthma. ACS Nano.

[12] Bianco A, Cheng HM, Enoki T, Gogotsi Y, Hurt RH, Koratkar N, Kyotani T, Monthioux M, Park CR, Tascon JMD, Zhang J (2013). All in the graphene family - a recommended nomenclature for two-dimensional carbon materials. Carbon.

[13] Wick P, Louw-Gaume AE, Kucki M, Krug HF, Kostarelos K, Fadeel B, Dawson KA, Salvati A, Vázquez E, Ballerini L (2014). Classification framework for graphene-based materials. Angew Chem Int Ed.

[14] Porter D, Sriram K, Wolfarth M, Jefferson A, Schwegler-Berry D, Andrew ME, Castranova V (2008). A biocompatible medium for nanoparticle dispersion. Nanotoxicology.

[15] Sanchez VC, Jachak A, Hurt RH, Kane AB (2012). Biological interactions of graphene-family nanomaterials: an interdisciplinary review. Chem Res Toxicol.

[16] Nolan A, Naveed B, Comfort AL, Ferrier N, Hall CB, Kwon S, Kasturiarachchi KJ, Cohen HW, Zeig-Owens R, Glaser MS (2012). Inflammatory biomarkers predict airflow obstruction after exposure to World Trade Center dust. Chest.

[17] Payne JP, Kemp SJ, Dewar A, Goldstraw P, Kendall M, Chen LC, Tetley TD (2004). Effects of airborne world trade center dust on cytokine release by primary human lung cells in vitro. J Occup Environ Med.

[18] Erdely A, Dahm M, Chen BT, Zeidler-Erdely PC, Fernback JE, Birch ME, Evans DE, Kashon ML, Deddens JA, Hulderman T, et al. Carbon nanotube dosimetry: From workplace exposure assessment to inhalation toxicology. Part Fibre Toxicol. 2013;10:53. 10.1186/1743-8977-10-53 PMC4015290 24144386

[19] Lo L-M, Hammond D, Bartholomew I, Almaguer D, Heitbrink W, Topmiller J. Engineering Controls for Nano-Scale Graphene Platelets During Manufacturing and Handling Procedures. Division of Applied Research and Technology, National Institute for Occupational Safety and Health, Cincinnati, OH; 2011.

[20] Porter DW, Hubbs AF, Mercer RR, Wu N, Wolfarth MG, Sriram K, Leonard S, Battelli L, Schwegler-Berry D, Friend S (2010). Mouse pulmonary dose- and time course-responses induced by exposure to multi-walled carbon nanotubes. Toxicology.

[21] Grosse Y, Loomis D, Guyton KZ, Lauby-Secretan B, El Ghissassi F, Bouvard V, Benbrahim-Tallaa L, Guha N, Scoccianti C, Mattock H (2014). Carcinogenicity of fluoro-edenite, silicon carbide fibres and whiskers, and carbon nanotubes. Lancet Oncol.

[22] Sargent LM, Porter DW, Staska LM, Hubbs AF, Lowry DT, Battelli L, Siegrist KJ, Kashon ML, Mercer RR, Bauer AK, et al. Promotion of lung adenocarcinoma following inhalation exposure to multi-walled carbon nanotubes. Part Fibre Toxicol. 2014;11:3. 10.1186/1743-8977-11-3 PMC3895742 24405760

[23] Dahm MM, Evans DE, Schubauerberigan MK, Birch ME, Fernback JE (2012). Occupational exposure assessment in carbon nanotube and nanofiber primary and secondary manufacturers. Ann Occup Hyg.

[24] Cassel SL, Joly S, Sutterwala FS (2009). The NLRP3 inflammasome: a sensor of immune danger signals. Semin Immunol.

[25] Hamilton RF, Wu N, Xiang C, Li M, Yang F, Wolfarth M, Porter DW, Holian A. Synthesis, characterization, and bioactivity of carboxylic acid-functionalized titanium dioxide nanobelts. Part Fibre Toxicol. 2014;11:43. 10.1186/s12989-014-0043-7 PMC4237951 25179214

[26] Sager TM, Wolfarth MW, Andrew M, Hubbs A, Friend S, Chen TH, Porter DW, Wu N, Yang F, Hamilton RF, Holian A (2014). Effect of multi-walled carbon nanotube surface modification on bioactivity in the C57BL/6 mouse model. Nanotoxicology.

[27] Sun B, Wang X, Ji Z, Wang M, Liao YP, Chang CH, Li R, Zhang H, Nel AE, Xia T (2015). NADPH oxidase-dependent NLRP3 inflammasome activation and its important role in lung fibrosis by multiwalled carbon nanotubes. Small.

[28] Donaldson K, Murphy FA, Duffin R, Poland CA (2010). Asbestos, carbon nanotubes and the pleural mesothelium: a review of the hypothesis regarding the role of long fibre retention in the parietal pleura, inflammation and mesothelioma. Part Fibre Toxicol.

[29] Gatoo MA, Naseem S, Arfat MY, Dar AM, Qasim K, Zubair S (2014). Physicochemical properties of nanomaterials: implication in associated toxic manifestations. Biomed Res Int.

[30] Utembe W, Potgieter K, Stefaniak AB, Gulumian M. Dissolution and biodurability: Important parameters needed for risk assessment of nanomaterials. Part Fibre Toxicol. 2015;12:11. 10.1186/s12989-015-0088-2 PMC4410501 25927337

[31] Warheit DB, Reed KL, Sayes CM (2009). A role for nanoparticle surface reactivity in facilitating pulmonary toxicity and development of a base set of hazard assays as a component of nanoparticle risk management. Inhal Toxicol.

[32] Seabra AB, Paula AJ, De Lima R, Alves OL, Durán N (2014). Nanotoxicity of graphene and graphene oxide. Chem Res Toxicol.

[33] Bianco A (2013). Graphene: Safe or toxic? the two faces of the medal. Angewandte Chemie - International Edition.

[34] Materials ASoT (2002). Standard test method for metal powder specific surface area by physical adsorption.

[35] Seehra MS, Pavlovic AS (1993). X-Ray diffraction, thermal expansion, electrical conductivity, and optical microscopy studies of coal-based graphites. Carbon.

[36] Suresh Babu V, Seehra MS (1996). Modeling of disorder and X-ray diffraction in coal-based graphitic carbons. Carbon.

[37] Janzen EG, Blackburn BJ (1968). Detection and identification of short-lived free radicals by an electron spin resonance trapping technique [11]. J Am Chem Soc.

[38] Foucaud L, Wilson MR, Brown DM, Stone V (2007). Measurement of reactive species production by nanoparticles prepared in biologically relevant media. Toxicol Lett.

[39] Pal AK, Bello D, Budhlall B, Rogers E, Milton DK (2012). Screening for oxidative stress elicited by engineered nanomaterials: Evaluation of acellular DCFH assay. Dose-Response.

[40] Hussain S, Boland S, Baeza-Squiban A, Hamel R, Thomassen LCJ, Martens JA, Billon-Galland MA, Fleury-Feith J, Moisan F, Pairon JC, Marano F (2009). Oxidative stress and proinflammatory effects of carbon black and titanium dioxide nanoparticles: Role of particle surface area and internalized amount. Toxicology.

[41] Koike E, Kobayashi T (2006). Chemical and biological oxidative effects of carbon black nanoparticles. Chemosphere.

[42] Sauvain JJ, Deslarzes S, Riediker M (2008). Nanoparticle reactivity toward dithiothreitol. Nanotoxicology.

[43] Sauvain JJ, Rossi MJ, Riediker M (2013). Comparison of three acellular tests for assessing the oxidation potential of nanomaterials. Aerosol Sci Technol.

[44] Underwood EE (1970). Quantitative stereology.

[45] Hubbs AF, Sargent LM, Porter DW, Sager TM, Chen BT, Frazer DG, Castranova V, Sriram K, Nurkiewicz TR, Reynolds SH (2013). Nanotechnology: toxicologic pathology. Toxicol Pathol.

[46] Mercer RR, Scabilloni JF, Hubbs AF, Wang L, Battelli LA, McKinney W, Castranova V, Porter DW (2013). Extrapulmonary transport of MWCNT following inhalation exposure. Part Fibre Toxicol.

[47] Schmittgen TD, Livak KJ (2008). Analyzing real-time PCR data by the comparative CT method. Nat Protoc.

