# Supplementary material for: Evaluation of pulmonary and systemic toxicity following lung exposure to graphite nanoplates: a member of the graphene-based nanomaterial family
Source: Part Fibre Toxicol. 2016 Jun 21;13:34. doi: 10.1186/s12989-016-0145-5 (PMC4915050; doi:10.1186/s12989-016-0145-5)
Supplement: Supplementary file 1 — Images of cells recovered by BAL from animals exposed to DM or 40 μg of Gr20, Gr5, Gr1, or CB, at 4 h, 7 days, and 2 months post-exposure illustrating particle burden in macrophages from the alveolar region. (PDF 1663 kb) [file 12989_2016_145_MOESM1_ESM.pdf]

Figure S1

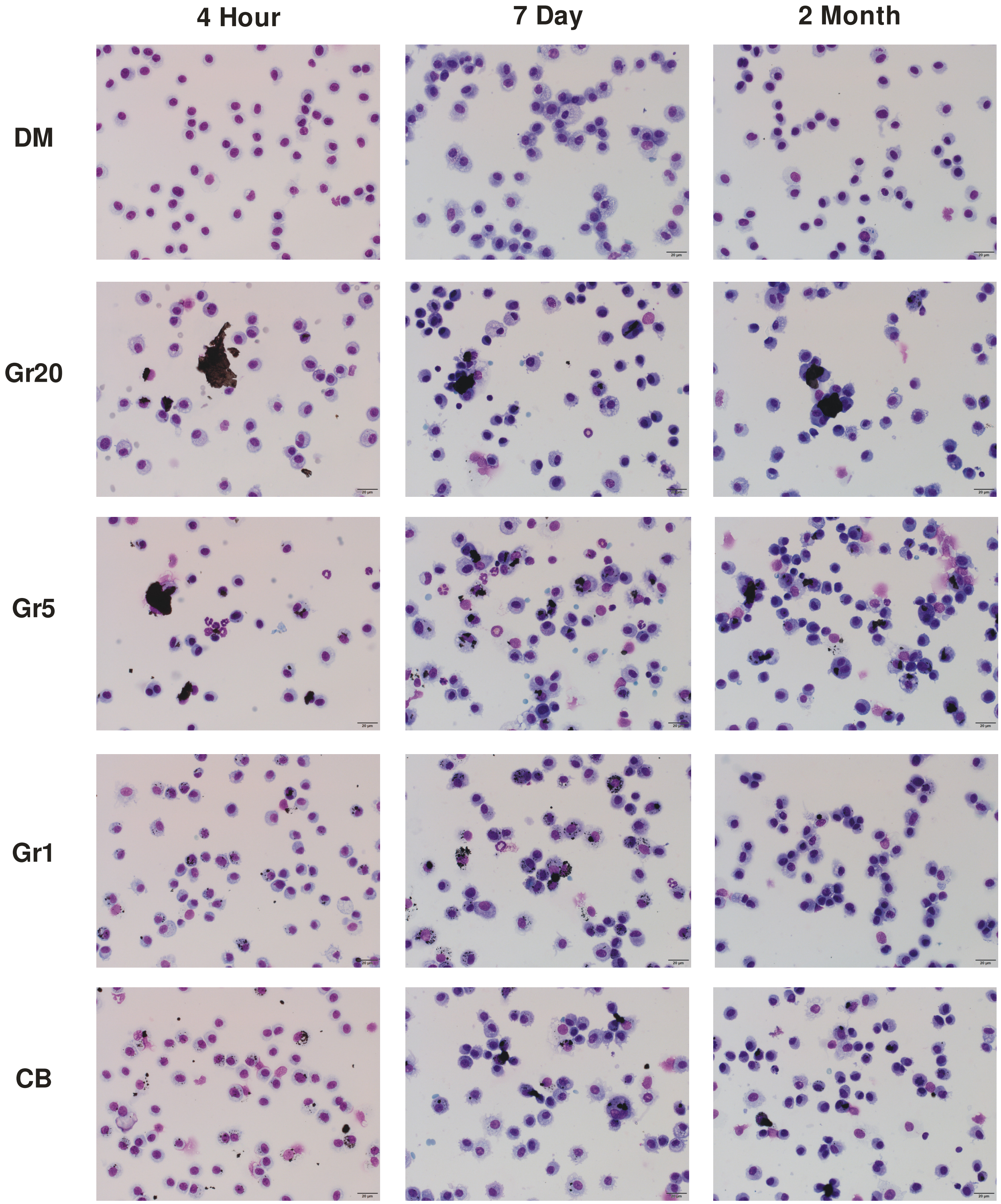

Supplemental Figure Legend: Images of cells recovered by BAL from animals exposed to DM or 40 μg of Gr20, Gr5, Gr1, or CB, at 4hours, 7 days, and 2 months post-exposure illustrating particle burden in macrophages from the alveolar region.
