# Supplementary material for: Evaluation of pulmonary and systemic toxicity following lung exposure to graphite nanoplates: a member of the graphene-based nanomaterial family
Source: Part Fibre Toxicol. 2016 Jun 21;13:34. doi: 10.1186/s12989-016-0145-5 (PMC4915050; doi:10.1186/s12989-016-0145-5)
Supplement: Supplementary file 2 — List of total proteins analyzed by RodentMAP® analyses, abbreviations, and alternate nomenclature. (PDF 32 kb) [file 12989_2016_145_MOESM2_ESM.pdf]

Table S1. List of total proteins analyzed by RodentMAP® analyses, abbreviations, and alternate nomenclature

1. Apolipoprotein A-1	Apo A-1
2. C-Reactive Protein Mouse	CRP Mouse
3. CD40	CD40; Bp50, TNFRSF5
4. CD40 Ligand	CD40L, CD154
5. Eotaxin	Eotaxin; CCL11 (eotaxin-1), CCL24 (eotaxin-2), CCL26 (eotaxin-3)
6. Epidermal Growth Factor Mouse	EGF Mouse; prostatric growth factor (PGF), urogastrone, human milk growth factor (HMGF)
7. Factor VII	Factor VII; proconvertin
8. Fibrinogen	Fibrinogen
9. Fibroblast Growth Factor-9	FGF-9; heparin binding growth factor (HBGF) - 9
10. Fibroblast Growth Factor-basic	FGF-basic
11. Granulocyte Chemotactic Protein-2 Mouse	GCP-2 Mouse; CXCL6, chemokine alpha (CKA)-3
12. Granulocyte-Macrophage Colony-Stimulating Factor	GM-CSF; CSF-2
13. Growth Hormone	GH; somatotropic hormone, somatotropin, somatropin
14. Growth-Regulated Alpha Protein	KC/GRO;CXCL1, neutrophil activating protein (NAP)-3, melanoma growth stimulating activity alpha (MGSA-α), cytokine induced neutrophil chemoattractant (CINC) – 2β
15. Haptoglobin	Haptoglobin
16. Immunoglobulin A	IgA
17. Insulin	Insulin
18. Interferon-gamma	IFN-γ; immune interferon (IIF), type 2 interferon, T-interferon, mitogen induced interferon
19. Interferon gamma Induced Protein 10	IP-10; CXCL10, C7
20. Interleukin-1 alpha	IL-1α
21. Interleukin-1 beta	IL-1β; catabolin
22. Interleukin-2	IL-2; lymphokine, T cell growth fractor (TCGF), lymphocyte proliferating factor (LPF)
23. Interleukin-3	IL-3; mast cell growth factor (MCGF), multi-CSF, hemopoietin-2, stem cell activating factor (SAF)

24. Interleukin-4	IL-4; B cell stimulating factor (BSF)-1, B cell growth factor (BCGF)-1
25. Interleukin-5	IL-5; BCGF-2, eosinophil differentiation factor (EDF)
26. Interleukin-6	IL-6; BSF-2, fibronectin stimulating factor (FSF), hepatocyte stimulating factor (HSF), natural killer activating factor (NKAF)
27. Interleukin-7	IL-7; pre-B cell growth factor (PBGF), lymphopoietin-1, thymocyte growth factor
28. Interleukin-10	IL-10; B cell derived T cell growth factor (B-TCGF), cytokine synthesis inhibitory factor (CSIF), T cell growth factor (TGIF)
29. Interleukin-11	IL-11; adipogenesis inhibitory factor (AGIF), megakaryocyte-CSF
30. Interleukin-12 Subunit p70	IL-12p70; cytotoxic lymphocyte maturation factor (CLMF), natural killer cell stimulatory factor (NKSF), T cell stimulatory factor (TSF)
31. Interleukin-17A	IL-17A
32. Interleukin-18	IL-18; IFN-γ inducing factor, IL-1γ, IL1F4
33. Leptin	Leptin; obesity factor, obese protein
34. Leukemia Inhibitory Factor	LIF
35. Lymphotactin	LTN; XCL1
36. Macrophage Colony-Stimulating Factor-1	M-CSF-1, CSF-1, macrophage growth factor (MGF)
37. Macrophage-Derived Chemokine	MDC, CCL22, stimulated T cell chemotactic protein (STCP)-1, dendritic cell/B cell derived chemokine (DC/B-Ck)
38. Macrophage Inflammatory Protein-1alpha	MIP-1α; CCL3
39. Macrophage Inflammatory Protein-1beta	MIP-1β; CCL4, endogenous pyrogen
40. Macrophage Inflammatory Protein-1gamma	MIP-1γ; CCL9, CCF18
41. Macrophage Inflammatory Protein-2	MIP-2, CXCL2, CINC-2α
42. Macrophage Inflammatory Protein-3 beta	MIP-3β, CCL19, EBI-1-Ligand chemokine (ELC), Exodus-3
43. Matrix Metalloproteinase-9	MMP-9;collagenase-5, gelatinase B, gelatinase type IV-B, polymorphonuclear leukocyte gelatinase
44. Monocyte Chemotactic Protein-1	MCP-1, CCL2, HC11, glioma-derived monocyte chemotactic factor (GDCF),
45. Monocyte Chemotactic Protein-3	MCP-3; CCL7, mouse mast cell activation-related chemokine (MARC)
46. Monocyte Chemotactic Protein-5	MCP-5; CCL12

47. Myeloperoxidase	MPO
48. Myoglobin	Myoglobin
49. Oncostatin-M	OSM
50. Plasminogen Activator Inhibitor 1	PAI-1; endothelial plasminogen activator inhibitor, mesosecrin, monocyte suppressor factor (MSF), serpin E1
51. Resistin	Resistin; adipose tissue-specific secretory factor (ADSF), C/EBP-epsilon-regulated myeloid-specific secreted cysteine-rich protein
52. Serum Amyloid P-Component	SAP
53. Stem Cell Factor	SCF; hemolymphopoietic growth factor (HLGF)-1, mast cell growth factor, stem cell growth factor (SCGF), kit ligand (KL)
54. T-Cell-Specific Protein RANTES	RANTES, CCL5, SIS-delta, eosinophil chemotactic polypeptide (EoCP)-1
55. Thrombopoietin	TPO; thrombopoeisis stimulating factor (TSF)
56. Tissue Inhibitor of Metalloproteinase 1 Mouse	TIMP-1; human gollaginase inhibitor (HCI), fibroblast elongation factor, fibroblast collagenase inhibitor beta-1 anticollaginase, embryogenin-1
57. Tumor Necrosis Factor alpha	TNF-α; cachectin, differentiation inducing factor (DIF), mono-derived fibroblast growth factor (MD-FGF)
58. Vascular Cell Adhesion Molecule-1	VCAM-1; CD106
59. Vascular Endothelial Growth Factor A	VEGF-A; vasculotropin, vascular, endothelial cell proliferation factor, vascular permeability factor (VPF)
60. von Willebrand factor	vWF
