# Supplementary material for: Evaluation of pulmonary and systemic toxicity following lung exposure to graphite nanoplates: a member of the graphene-based nanomaterial family
Source: Part Fibre Toxicol. 2016 Jun 21;13:34. doi: 10.1186/s12989-016-0145-5 (PMC4915050; doi:10.1186/s12989-016-0145-5)
Supplement: Supplementary file 3 — Genes contained in the pre-designed TaqMan® array. (PDF 22 kb) [file 12989_2016_145_MOESM3_ESM.pdf]

Table S2: **Genes contained in the pre-designed TaqMan® array**

Gapdh-Mm99999915_g1	Clu-Mm00442773_m1
Hprt-Mm01545399_m1	Fos-Mm00487425_m1
Il1a-Mm00439620_m1	Mt1-Mm00496660_g1
Il1b-Mm00434228_m1	Mt2-Mm00809556_s1
Il5-Mm00439646_m1	Fas-Mm00433237_m1
Il6-Mm00446190_m1	Hmox1-Mm00516004_m1
Il10-Mm00439616_m1	Serpine1-Mm00435860_m1
Il12a-Mm00434165_m1	Thbs1-Mm01335418_m1
Il13-Mm00434204_m1	F3-Mm00438853_m1
Ifng-Mm00801778_m1	Fgg-Mm00513575_m1
18S-Hs99999901_s1	Edn1-Mm00438656_m1
Tnf-Mm00443258_m1	Ednra-Mm01243722_m1
Ptgs2-Mm00478374_m1	Sele-Mm00441278_m1
Icam1-Mm00516023_m1	Selp-Mm00441295_m1
Arg1-Mm00475988_m1	Vcam1-Mm01320970_m1
Arg2-Mm00477592_m1	Pecam1-Mm00476702_m1
Itgam-Mm00434455_m1	Mmp9-Mm00442991_m1
Emr1-Mm00802529_m1	Mmp2-Mm00439498_m1
Nos2-Mm00440485_m1	Timp1-Mm00441818_m1
Ccl2-Mm00441242_m1	Timp2-Mm00441825_m1
Ccl4-Mm00443111_m1	Timp4-Mm00446568_m1
Ccl11-Mm00441238_m1	Col1a1-Mm00801666_g1
Ccl22-Mm00436439_m1	Col3a1-Mm00802331_m1
Ccl5-Mm01302428_m1	Col4a4-Mm00801574_m1
Ccr2-Mm99999051_gH	Col4a2-Mm00802386_m1
Ccr5-Mm01963251_s1	Nid1-Mm00477827_m1
Csf1-Mm00432688_m1	Nid2-Mm00456212_m1
Csf2-Mm00438328_m1	Igf1r-Mm00802831_m1
Csf3-Mm00438334_m1	Igf2-Mm00439565_g1
Cxcl1-Mm00433859_m1	Igf1-Mm00439561_m1
Cxcl2-Mm00436450_m1	Vegfa-Mm00437304_m1
Cxcl12-Mm00445552_m1	Pdgfa-Mm00435540_m1
Cxcr2-Mm99999117_s1	Fgfr1-Mm00438923_m1
S100a8-Mm00496696_g1	Pgap2-Mm00523992_g1
Spp1-Mm00436767_m1	Fgf1-Mm00438906_m1
Hspa1b-Mm03038954_s1	Egf-Mm00438696_m1
Dnaja1-Mm00787254_s1	Ctgf-Mm00515790_g1
Hsp90aa1-Mm00658568_gH	Tgfb1-Mm00441724_m1
Hif1a-Mm00468875_m1	Tgfbr1-Mm00436971_m1
Hif1an-Mm00621168_m1	Ltbp1-Mm00498234_m1
Hif3a-Mm00469373_m1	Sod2-Mm00449726_m1
Egr1-Mm00656724_m1	Vwf-Mm00550376_m1
Bbc3-Mm00519268_m1	Cd44-Mm01277163_m1
Casp1-Mm00438023_m1	Nfe2l2-Mm00477784_m1
Casp4-Mm00432307_m1	Cat-Mm00437992_m1
Gadd45a-Mm00432802_m1	Nqo1-Mm00500821_m1
Cryaa-Mm00660256_m1	Cyba-Mm00514478_m1
Rrad-Mm00451053_m1	Nox4-Mm00479246_m1
