# Supplementary material for: Evaluation of pulmonary and systemic toxicity following lung exposure to graphite nanoplates: a member of the graphene-based nanomaterial family
Source: Part Fibre Toxicol. 2016 Jun 21;13:34. doi: 10.1186/s12989-016-0145-5 (PMC4915050; doi:10.1186/s12989-016-0145-5)
Supplement: Supplementary file 4 — Differentially expressed genes in lung tissue following exposure to 40 μg of Gr20, Gr5, or Gr1. (PDF 38 kb) [file 12989_2016_145_MOESM4_ESM.pdf]

Table S3. Differentially expressed genes in lung tissue following exposure to 40 µg of Gr20, Gr5, or Gr1.

	4 Hour			1 Day		
**Gene**	**Gr 20 - High**	**Gr 5 - High**	**Gr 1 - High**	**Gr 20 - High**	**Gr 5 - High**	**Gr 1 - High**
Il1b	↑	↑↑	↑	↑	↑	
Il5	↑↑↑	↑↑↑	↑↑	↑	↑	
Il6	↑↑↑	↑↑↑	↑↑↑	↑↑↑	↑↑	↑
Il10	↑	↑↑				
Il13	↑↑↑	↑↑↑	↑↑			
Arg1	↑	↑		↑↑	↑↑	
Arg2				↑	↑	
Ccl2	↑↑↑	↑↑↑	↑↑↑	↑↑↑	↑↑↑	↑↑
Ccl11	↑↑	↑↑↑	↑	↑↑	↑↑	
Ccl22	↑↑	↑↑	↑	↑	↑	
Ccr5		↑↑				
Csf2					↑	
Csf3	↑↑↑	↑↑↑	↑↑↑			
Cxcl1	↑↑	↑↑↑	↑↑↑	↑↑↑	↑↑↑	↑↑
Cxcl2	↑↑	↑↑↑	↑↑↑	↑↑	↑↑	↑↑
Cxcr2		↑				
Hspa1b	↑	↑↑				
Bbc3		↓				
Casp4	↑	↑	↑	↑	↑	
Cryaa	↓	↓				
Fos	↑	↑	↑			
Mt1	↑↑	↑↑	↑	↑		
Mt2	↑↑	↑↑↑	↑↑			
Hmox1				↑		
Serpine1	↑	↑↑	↑			
Thbs1	↑	↑	↑			
Fgg				↑↑	↑	
Sele	↑↑↑	↑↑↑	↑↑↑			
Selp	↑↑↑	↑↑↑	↑↑↑	↑		
Timp1	↑↑	↑↑	↑↑	↑↑	↑↑	↑
Timp4				↑		
Igf1				↑	↑	
Cat		↓				
Nox4		↓				

Note: A single arrow indicates a 1.5-3.5 fold change, a double arrow indicates a 3.6-10 fold change, and a triple arrow indicates a 10 fold change.
